# Beyond stone and timber: the connotative meaning of built heritage, its associations with personal values and the mediating role of time orientations

**DOI:** 10.3389/fpsyg.2026.1903386

**Published:** 2026-08-26

**Authors:** Barbara Fogarasi, Iván Zsolt Berze, Ágnes Buvár, Andrea Dúll

**Affiliations:** 1 Doctoral School of Psychology, ELTE Eötvös Loránd University, Budapest, Hungary; 2 Institute of People–Environment Transaction, Faculty of Education and Psychology, ELTE Eötvös Loránd University, Budapest, Hungary; 3 Department of Sociology and Communication, Budapest University of Technology and Economics, Budapest, Hungary

**Keywords:** built heritage, connotative meaning, path analysis, personal values, person-environment transaction, semantic differential, time perspective

## Abstract

Although people associate values with their experiences of heritage places, these are often overlooked in the preservation and reuse of historic buildings and sites. Little is known about how meanings are attributed to built heritage and how they relate to broader psychological constructs. This study explored connotative meaning using a semantic differential scale developed for built heritage, identifying three dimensions—evaluation, human connectedness, and condition—and investigated their relationships with personal values (Schwartz's Value Portrait Questionnaire) and time orientations (Zimbardo's Time Perspective Inventory). Path analysis revealed that self-transcendence and openness to change values are directly and positively associated with all the identified connotative meaning dimensions of built heritage. In contrast, self-enhancement values are directly negatively associated with all meaning dimensions. Additionally, past-positive time orientation totally mediates the positive link between conservation values and the evaluation dimension, while future time orientation totally mediates the positive relationship between conservation values and the condition dimension. Age is positively associated with all built heritage meaning dimensions. The findings bring us closer to understanding the relationship between heritage meanings, value systems, and time perspectives, informing sustainable heritage management and its role in enhancing the quality of life.

## 1 Introduction

There is increasing empirical evidence in environmental psychology that built heritage has transactional relationships with individual and collective identity and memory (Light and Dumbraveanu-Andone, 1997; Lewicka, 2008; Goulding and Domic, 2009; Belanche et al., 2017; Ramirez and Benali, 2018; Fogarasi and Dúll, 2021; Gantois, 2021; Yang et al., 2022), and is also associated with mental wellbeing (Sofaer et al., 2021; Gallou et al., 2022; Richardson et al., 2025; Sherborne and Malthouse, 2026). Experiencing historic environments may increase positive affect and reduce anxiety (Grossi et al., 2019; Scopelliti et al., 2019; Gallou et al., 2022), while engagement with physical heritage improves mental health (Heaslip et al., 2020). Several studies highlight the restorative effects of historic places (Ouellette et al., 2005; Bornioli et al., 2018; Li et al., 2023; Wang et al., 2023; Miola et al., 2026), and an expanding body of place attachment literature examines the intricate relationship between people and built historic environments (Lewicka, 2011; Lewicka et al., 2023; Scannell and Gifford, 2010; Stedman, 2002; Twigger-Ross and Uzzell, 1996; Wang, 2023). Research indicates that the appreciation for both built and natural environments increases with age, as older adults often report higher levels of place attachment, environmental satisfaction, and positive evaluations of their surroundings compared to younger people (Giuliani, 2003; Hernández et al., 2007; Lewicka, 2011). Visiting heritage sites is positively related to life satisfaction (Fujiwara et al., 2014), and out of the heritage categories, historic towns and historic buildings are found to have the most positive influence on subjective wellbeing (Sektani et al., 2023). Studies have also indicated associations between heritage tourism experiences and local heritage connectedness with pro-environmental behaviors (Wu et al., 2020; Fu and Dong, 2025; Richardson et al., 2025). Built heritage is increasingly seen as contributing to environmental sustainability through conservation actions, like renovating and retrofitting historic structures, which enhance energy efficiency, reduce carbon emissions, and promote environmentally conscious behavior (Behbehani and Prokopy, 2017; Wise et al., 2021; Lerario, 2022; Gonçalves et al., 2023; Shah et al., 2023; Jensen et al., 2025).

Despite these known beneficial relationships of built heritage with people's psychological processes and wellbeing, as well as the protection of the environment, the survival and upkeep of built heritage remain challenging. Safeguarding buildings and sites of historical significance for present and future generations is considered to be primarily the task of built heritage preservation professionals. At the same time, their long-term care depends much on communities, individuals, and decision makers, who will only engage in maintenance and restoration efforts if they recognize and appreciate the benefits, which are not always self-evident (Fogarasi et al., 2017). While heritage professionals have established methods for assessing expert significance and value, much less is known about how laypeople experience and evaluate historic places. Among studies on the transactional relationship between people and their socio-physical environment (Stokols, 1978), the attitudes, values, and connotative meaning associated with built historic environments remain underexplored. In particular, little is understood about the connotative meaning dimensions people assign to such places or how individual differences shape them. To bridge this gap, conservation and adaptive reuse efforts must draw on insights from environmental psychology to better understand the psychological factors influencing public engagement with built heritage. Pro-environmental behavior is often associated with the protection of natural environments, yet it may also be understood to include the built environment and, within it, heritage preservation. From an environmental psychology perspective, both natural and man-made settings carry meanings that are interrelated with identity, values, and wellbeing. In this sense, caring for historic places can be seen as reflecting similar psychological orientations and a collective responsibility for sustaining our surroundings that also motivate pro-environmental action. Recognizing the reciprocal relationship between people and historic places is essential: heritage sites depend on human involvement for their protection and sustainability, while in turn they shape people's identity, social cohesion, and wellbeing, which again influences how people assign meaning to them.

The current study forms part of a broader investigation on the psychological aspects of built historic places, including the development of a tool to measure the connotative meaning of built heritage (Fogarasi et al., 2023) and comparisons of lay and expert assessments (see Appendix A). The present paper focuses on individual differences, specifically, examining how personal values (PVs) and time perspectives (TPs) relate to the connotative meaning of built heritage. Considering previous findings on the relationships between PVs and TPs (Maciuszek et al., 2019; Kawakami et al., 2020), path analysis was conducted to examine the relationship patterns between the revealed built heritage meaning dimensions (BHMDs), PVs, TPs, and age, controlling for gender. The analysis also allowed testing whether associations previously observed in research on pro-environmental attitudes are reflected in the connotative meanings attributed to the built historic environment, while acknowledging that attitudes and meaning attribution represent related but distinct constructs. Furthermore, we examined whether TPs mediate the associations of BHMDs with PVs and age. We assumed that values influence built heritage assessment via TPs, because, as suggested by other research (Maciuszek et al., 2019), values are more fundamental and stable. According to the aims and based on the literature findings discussed in the next section, we formulated four explorative research questions (RQ) and within these, five hypotheses (H) as follows:

RQ1: We aimed to reveal the (direct) associations between personal values and built heritage meaning dimensions. In this frame, it was expected that, independent of time perspectives,

H1: self-enhancement values would negatively relate to built heritage meaning dimensions, and
H2: self-transcendence values would positively relate to built heritage meaning dimensions.

RQ2: We also aimed to uncover the associations between time perspectives and built heritage meaning dimensions. In this frame, it was expected that

H3: past-positive time perspective would be positively associated with built heritage meaning dimensions,
H4: present-fatalism time perspective would be negatively associated with built heritage meaning dimensions, and
H5: future orientation would be positively associated with built heritage meaning dimensions.

RQ3: Our goal was to reveal the associations between age and built heritage meaning dimensions.

RQ4: Finally, we intended to investigate whether time perspectives mediate the associations of personal values and age with built heritage meaning dimensions.

**Figure 1:**
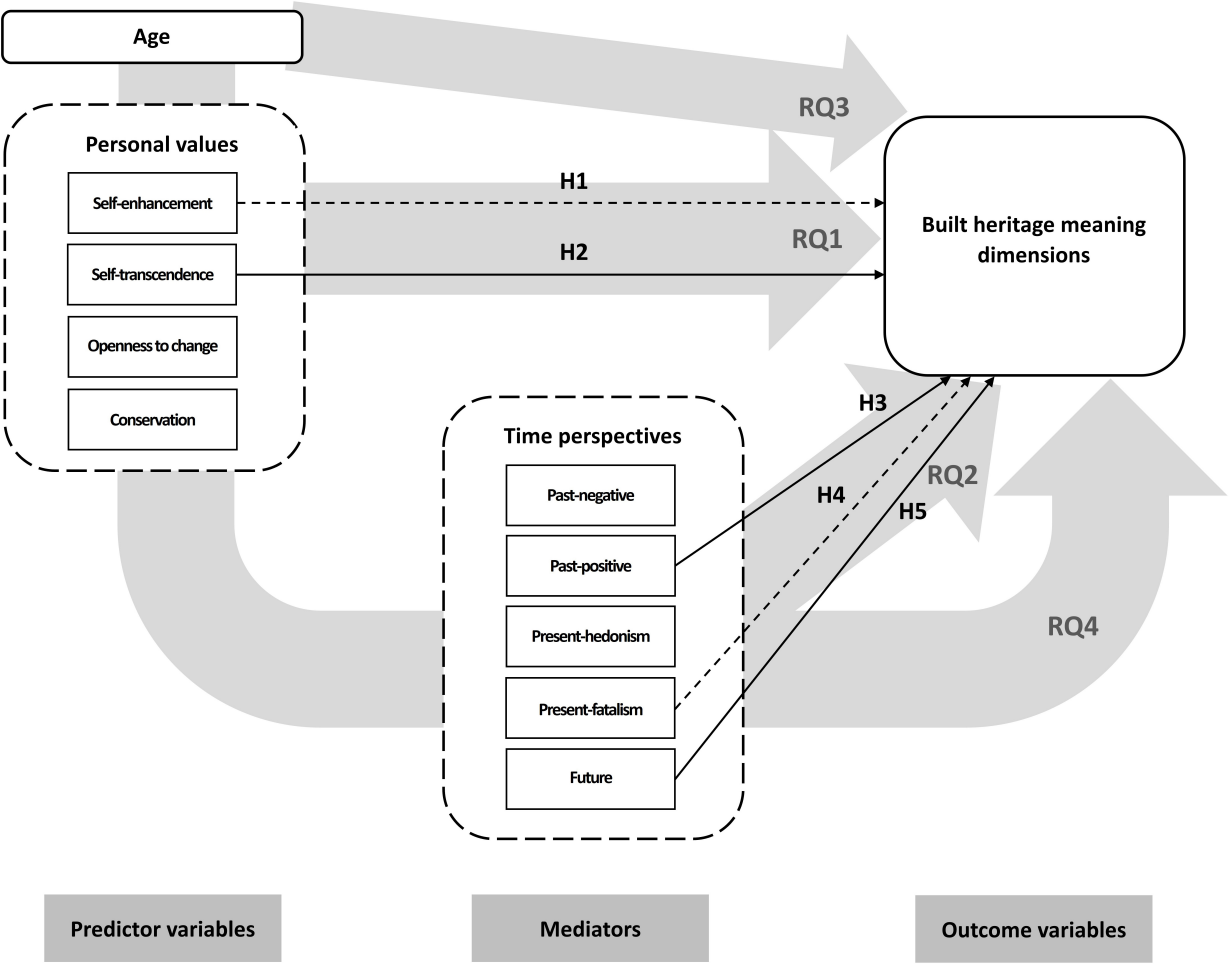
Conceptual model.

The conceptual research model is illustrated in Figure 1.

## 2 Literature review

### 2.1 The social value of built heritage

While various long-established value-based assessment approaches exist in the heritage preservation field (see a summary of 20th century heritage value typologies devised by various scholars and organizations in De La Torre, 2002, p. 9.) to identify categories of values that make up “cultural significance,” the inclusion of “social value” is inconsistent (Avrami et al., 2000; Gibson and Pendlebury, 2009; De La Torre, 2013; Fredheim and Khalaf, 2016; Shaheen and Metawi, 2022). Although there is no single accepted definition, the concept captures the relationship between the historic environment and people's sense of belonging, identity, distinctiveness, wellbeing, memory, spiritual association and cultural practice, often refering to concepts like attachment to place, symbolic value, spiritual associations, social capital, and even recreation and education (Jones and Leech, 2015). Social, communal, spiritual, symbolic, and emotional values are inherently more fluid and interpretive than objective, intrinsic historic, scientific or architectural ones, making them difficult to identify and continually subject to redefinition in national and international frameworks (Chen and Wang, 2024). This results in partial assessments often relying on anecdotal evidence (Jones and Leech, 2015). Resource limitations and competing demands within heritage organizations frequently hinder the systematic investigation of social value in conservation and management practices. While international guidelines acknowledge intangible dimensions and the importance of meaning (ICOMOS Australia, 1979), social values are still largely determined by heritage professionals, reflecting what Smith (2006) terms the “Authorized Heritage Discourse,” driven by expert values. Even when guidelines are developed and followed to identify the different aspects of social value that should be considered or when community surveys are conducted to capture people's perceptions, experiences, or memories of a site, the input is often treated as raw data and interpreted through a professional lens before being incorporated into value assessments. Such practices raise concerns about whether social value authentically reflects the fluidity of personal heritage experience (Richardson et al., 2025) and the bonds people form with these places. Moreover, social values are dynamic and evolving, as users may change, which necessitates ongoing reassessment (Chen and Wang, 2024). Qualitative and participatory research methods derived from sociology and anthropology provide a deeper understanding of how people relate to the environment in ways that contribute to the formation of social value (Jones, 2017; Robson, 2025). Incorporating people's values into decisions about the conservation, restoration, and adaptive reuse of built heritage requires actively engaging stakeholders and local communities through collaborative methods (Skoglund and Svensson, 2010; Van Balen and Vandesande, 2015; Fogarasi et al., 2016). Participatory approaches enable communities to contribute their perspectives, needs, and values, fostering genuinely inclusive outcomes. Community-based methods, such as ICCROM's Living Heritage Approach (Poulios, 2014), actively involve communities to identify, appreciate, and sustain both tangible and intangible heritage values. The present findings complement existing approaches to understanding social value by exploring the connotative meaning dimensions through which people perceive, interpret, and experience built heritage sites. The identified *evaluation* meaning dimension refers to value-based judgements and affective appraisals, while *human connectedness* is consistent with transactional perspective of environmental psychology between people and places, and the *condition* dimension reflects perceptions of the physical state, which may be linked to conservation-related concepts such as integrity.

### 2.2 Exploring the connotative meaning of built heritage using the semantic differential

The meaning dimensions of human thought have long been a focus of research, notably by Osgood et al. (1957), who proposed that the meaning of any phenomenon can be conceptualized within a “semantic space” defined by a limited number of dimensions. To measure connotative meaning and assess the direction of value judgments based on emotional reactions and perceptions of any object or concept, the semantic differential scale was developed. The three most prominent dimensions found were *evaluation* (good/bad), *potency* (strong/weak), and *activity* (active/passive). These dimensions reflect fundamental human experiences and are deeply intertwined with core aspects of the meaning of human life. In this respect, numerous efforts were made to describe the multi-layered meanings embedded in the physical environment (Vielhauer, 1965; Alexander et al., 1978; Genereux et al., 1983; Krampen, 1991). The semantic differential technique appears in several studies assessing environmental perceptions and preference, as well as to evaluate the applicability of Osgood's three primary dimensions in describing the connotative meaning of socio-physical environments (Canter, 1969; Hershberger and Clements, 1969; Küller, 1972; Gärling, 1976; Horayangkura, 1978; Pedersen, 1978; Dúll and Urbán, 1997; Bishop, 2007; Markovic and Alfirevic, 2015; Ng, 2020; Guo et al., 2026). Despite these efforts, Osgood's dimensions have not been definitively validated in this context, and the quest for the most applicable and generalizable dimensions remains ongoing. In Hungary, (Dúll and Urbán 1997) developed an environmental semantic differential scale to describe the quality of environment-behavior interactions and to investigate connotative meaning of university environments, later used in other contexts, producing different meaning dimensions. Previous studies have thus repeatedly demonstrated that the meaning dimensions elicited by the method vary according to the type of environment and the characteristics of the respondents. Accordingly, the present study focuses on identifying the connotative meaning—understood as affective meaning elicited in response to environmental triggers—specific to built heritage. Given that attitudes toward the environment, including the historic built environment, are shaped by processes that are often not conscious, the semantic differential scale serves as a useful tool for uncovering and comparing users' affective relationships. The tool helps illustrate how meaning is defined within a given population, as interpretations of the built environment are influenced by cultural background, personal experiences, and expertise. Since meaning is inherently population-dependent, variations in perception can be observed across different groups, as revealed in our research on the connotative meaning attributed to historic built environments by heritage experts and laypeople (Fogarasi et al., 2023). We identified a higher number of meaning dimensions among professionals, indicating that they have a more complex and refined understanding of the subject.

To capture people's emotive meanings related to built historic environments, it is essential to understand how meanings are formed and represented. Meaning has been described as “associational, sociocultural qualities encoded into environmental elements, characteristics, or attributes” that influence “emotions, attitudes, preferences, and behavior” Rapoport, 1990, pp. 38–39). Connotative meaning, as conceptualized by (Osgood et al. (1957), refers to the affective and evaluative meaning that individuals attribute to objects, concepts or places, through experience and interaction. From the transactional perspective of environmental psychology, such meaning emerges from the dynamic relationship between people and environments. These meaning attributions are closely related to attitudes, shaped by the concepts and beliefs that develop through experience and interaction with particular places or categories of places (Casakin and Kreitler, 2008). Psychological approaches to meaning further suggest that individuals organize complex environmental information into mental representations of attributes and their interrelations (Dúll, 2009). Mapping these representations through tools such as the semantic differential enables the identification of latent meaning dimensions and their associations with individual characteristics, offering deeper insight into how people perceive and interpret built historic environments.

### 2.3 Personal values and time perspectives

In addition to identifying the connotative meaning attributed to built heritage, we explored how individual differences might shape this meaning. Specifically, we examined psychological orientations related to values and time, both being universal human factors that influence attitudes shaped by meaning, to assess their relation to perceptions of the historic built environment, comprising objects of the past that exist in the present and endure into the future. While personal values guide and motivate evaluations and behavior (Maio et al., 2001; Bardi and Schwartz, 2003; Boer and Fischer, 2013), time orientations are also associated with attitudes and actions (Baird et al., 2020; Daugherty and Brase, 2010; Strathman et al., 1994). Yet, research on how personal values and time perspectives relate to meanings and attitudes toward built heritage remains scarce.

Rokeach (1973) defines personal values as socially or personally preferred life paths or purposes of existence, describing them as “the main independent variable in the study of social attitudes and behavior” (Rokeach, 1973, p. ix). Elaborating on Rokeach, Schwartz (Schwartz and Bilsky, 1987; Schwartz, 1994) describes values as beliefs closely connected with affect (Schwartz, 2012). His theory of basic human values identifies 10 value types, each representing a broad goal driven by different individual or social motivations. These values are classified into four dimensions: *self-enhancement* (achievement and power), *openness to change* (hedonism, stimulation, and self-direction), *self-transcendence* (universalism, benevolence), and *conservation* (security, conformity, and tradition). Schwartz's typology is widely adopted in environmental psychology, particularly for assessing environmental attitudes and behavior (Stern et al., 1995; Karp, 1996; Follows and Jobber, 2000; Schultz et al., 2005; Milfont and Gouveia, 2006; De Groot and Steg, 2008; De Groot et al., 2012; Boer and Fischer, 2013; Marshall et al., 2019; Tan et al., 2022; Eylering et al., 2025). Most of these studies found that self-transcendence or biospheric values positively correlate, and self-enhancement or egotistic values negatively correlate with conservation and pro-environmental attitudes and behavior. Preservation of tradition and customs is positively associated with altruistic values and negatively with egoistic values (Megeirhi et al., 2020).

People's unique perspectives on the future, memories of the past, and present demands affect temporal priorities, which influence important psychological processes, i.e., decision-making (Karniol and Ross, 1996; Milfont and Demarque, 2015; Stolarski et al., 2015). Zimbardo and Boyd introduced the Zimbardo Time Perspective Inventory (ZTPI, Zimbardo and Boyd, 1999), one of the most widely validated scales globally for assessing temporal orientations. The ZTPI identifies five distinct perspectives: *past-positive*, characterized by warm, nostalgic, and favorable perception of the past; *past-negative*, marked by negative, pessimistic, or aversive attitudes toward past experiences; *present-hedonistic*, emphasizing pleasure-seeking, risk-taking and enjoyment in the present moment; *present-fatalistic*, reflecting a sense of helplessness or hopelessness about the future and life in general; and *future*, which denotes a forward-thinking mind-set focused on setting and achieving goals. The scale has been extensively used in research on environmental perceptions, attitudes, and behavior. The relationships between time perspective and pro-environmental behavior were found to be stronger than those with pro-environmental attitudes (Milfont et al., 2012). Studies indicate that pro-environmentalism is primarily linked to a future-oriented perspective and, to a lesser extent, to past-positive orientation (Strathman et al., 1994; Corral-Verdugo et al., 2006; Milfont and Gouveia, 2006; Milfont et al., 2012; Carmi and Arnon, 2014; Stolarski et al., 2018; Ágoston et al., 2023; Tucholska et al., 2024). In contrast, present-fatalist orientation is associated with lower pro-environmental behavior (Stolarski et al., 2018).

The proposed hypotheses of the present research are informed by these previous studies on environmental attitudes, as concern for built heritage can also be understood as a form of environmental responsibility through the preservation and adaptive reuse of existing buildings. At the same time, built heritage differs from natural environments in several important aspects, including its human-made character, symbolic significance, and historical layers, which may result in distinct patterns of associations with personal values and time perspectives. The connotative meaning dimensions identified in this study capture the ways in which people relate to heritage places; therefore, we expected that value orientations previously linked to environmental concern might also be associated with the ways people relate to built heritage, while acknowledging the specific characteristics of heritage contexts. Specifically, self-transcendence values were expected to be positively associated with built heritage meanings, particularly with the *evaluation* dimension, reflecting appreciation of the significance and continuity of heritage places, and with the *human connectedness* dimension, as this reflects relational experiences involving people, objects and places. Conversely, self-enhancement values were expected to show weaker or negative associations with these dimensions. Likewise, past-positive and future-oriented perspectives were expected to be positively associated with the appreciation of historically embedded places and their continuity over time, whereas a present-fatalistic perspective was expected to be negatively associated with heritage meanings that involve continuity and personal engagement, given its emphasis on limited personal control over future outcomes.

## 3 Materials and methods

### 3.1 Statement of ethical practice

Ethical approval was obtained from the Research Ethics Committee of the Faculty of Education and Psychology, ELTE Eötvös Loránd University, Budapest, No. 2021/282-2 (October 20, 2021). The study was conducted in accordance with the ethical principles of the Declaration of Helsinki and relevant institutional guidelines. Informed consent was obtained electronically before completing the questionnaire. Participants received information about the aims and procedures of the study, the voluntary and anonymous nature of participation, and their right to withdraw at any time.

### 3.2 Design and participants

A questionnaire-based study was conducted online in Hungarian between November 2021 and May 2022, using Qualtrics software. The survey was distributed via social media (Facebook, without the use of paid advertisements), relying on organic sharing within social networks. In addition, the survey was integrated into two undergraduate university courses, where students received course credit for completing the questionnaire and for sharing the survey invitation with family and friends through their personal networks. In the two course cohorts, 36 and 45 students participated, respectively. While the exact proportion of student-recruited participants in the final sample cannot be determined, a total of 162 respondents self-identified as students or university students. Altogether 434 people completed the survey, which included an open-ended question on participants' profession and included a specific question on whether they were heritage professionals. Three respondents identified as heritage professionals and were excluded from the analysis, yielding a final sample of 431 laypeople, consisting of 64% women (*M*_age_ = 33.61 years, SD_age_ = 15.32, min: 19 years, max: 84 years). Response rates and non-response bias were not calculated, as these indicators were not considered meaningful within the present study design.

### 3.3 Measures

In addition to demographic questions, such as age, gender, education, profession, and place of residence, the questionnaire included the following measures:

#### 3.3.1 Semantic differential scale developed for built heritage

To measure the built heritage meaning dimensions, we used a 30-item semantic differential scale for built heritage (SDBH), a revised version of the 40-item scale developed in the preliminary study (Fogarasi et al., 2023; see Appendix A). The final 30-item version was established based on the PCA results of the initial scale, through an evaluation of item–factor loadings, inter-item correlations, and expert judgement. The visual stimuli consisted of five 360° panoramic images, the same as those used in the preliminary study, selected by three heritage experts and representing different heritage types: a ruin, a historic urban square, vernacular architecture, a symbolic monument, and an industrial heritage site. The images were obtained from Google Maps, with the corresponding links provided in the Supplementary Material (Appendix A). Images represented each heritage site from a pedestrian's perspective, approximately at eye level, thereby taking up the viewpoint of an on-site visitor. During image selection, care was taken to ensure comparable visual quality and lighting conditions whenever possible. All selected sites were in a generally well-maintained condition. The ruin site represented the characteristics of historical remains, including visible material aging and fragmentation, but did not display neglect or unsafe deterioration. Interior panoramic views were used for the vernacular type (a folk house) and the industrial type (a power plant), reflecting the typical visitor experience at these sites. The symbolic monument was presented as an illuminated evening scene because this is its most iconic view. The presence of other visitors was not used as an exclusion criterion, because the aim was to capture heritage sites as they are typically experienced by visitors; therefore, some images included other people in the environment, while others did not. The images were presented in a randomized order for each participant, and as such, the sequence of image presentation varied between participants to minimize potential order effects. Before viewing each image, participants were instructed to imagine themselves in the space, explore the details, immerse themselves in the environment, and then complete the SDBH for the particular site. Viewing time was not restricted; participants were allowed to inspect each panoramic image for as long as they needed to more closely resemble on-site visual exploration; however, they were not allowed to revisit previously viewed images to minimize direct comparisons across heritage sites and to ensure that ratings were based on their initial impressions. After viewing each image, items were rated on a 7-point scale, from 1 (left-side adjective, e.g. exciting) to 7 (right-side adjective, e.g. boring). Based on PCA results, the scale comprised three components, interpreted as *evaluation, human connectedness*, and *condition*. Adjective pairs were reversed as needed according to the sign of their factor loadings, so that higher scores consistently reflected the positive interpretive direction of each component, enhancing clarity for regression analyses (e.g., the “ornate–unadorned” pair was coded with “ornate” as 7). As all retained items had factor loadings above 0.50, the three meaning dimension scores were calculated using simple summation of the responses to the semantic differential items loading on each factor for each heritage site. Mean scores across the five heritage sites were then calculated for each meaning dimension to obtain an overall representation of each participant's responses. Table 1 displays the minimum and maximum achievable scores, number of items, and example items for the three subscales. Higher scores on each meaning dimension indicate greater perceived value, stronger human connectedness, and higher completeness attributed to the particular heritage site and also heritage sites in general. The SDBH was designed as a semantic differential tool aimed at capturing the multidimensional and context-dependent connotative meaning structures associated with the complex domain of built heritage in a relational framework. This implies that some variability in factor structure may occur across different heritage contexts, populations, and evaluative settings.

**Table 1:** Built heritage meaning dimensions resulting from the principal component analysis.

Meaning dimension	Theoretically achievable min.-max. scores	No. of items	Item example
Evaluation	10–70	10	special–ordinary
Human connectedness	9–63	9	intimate–cold
Condition	4–28	4	ruined–intact

#### 3.3.2 Schwartz's Portrait Values Questionnaire (PVQ)

One of the instruments developed by Schwartz to measure personal values is the Portrait Values Questionnaire (PVQ, Schwartz et al.'s, 2001). Participants completed the Hungarian version of the 21-item PVQ-21, adapted for the European Social Survey (Schwartz, 2003). Each item in the questionnaire portrays the goals and priorities of other persons, and participants rated how similar they are to these individuals on a 6-point Likert scale (1 = Not like me at all, to 6 = Very much like me). The PVQ measures 10 basic values organized into four higher-order value categories as shown in Table 2, displaying the minimum and maximum reachable scores, the number of items, and example items for each subscale. Higher scores indicate stronger higher-order personal value in all cases.

**Table 2:** Value categories in Schwartz's Portrait Value Questionnaire (PVQ, Schwartz, 2003).

Higher-order value	Theoretically achievable min.–max. scores	Basic value	No. of items	Item example
Self-enhancement	4–24	Power	2	“It is important to them to be in charge and tell others what to do. They want people to do what they say”
		Achievement	2	“Being very successful is important to them. They hope people will recognize their achievements”
Self-transcendence	5–30	Benevolence	2	“It's very important to them to help the people around them. They want to care for others' wellbeing”
		Universalism	3	“Looking after the environment is important to them”
Openness to change	6–36	Hedonism	2	“Having a good time is important to them. They like to “spoil” themselves”
		Stimulation	2	“They look for adventures and likes to take risks. They want to have an exciting life”
		Self-direction	2	“It is important to them to make their own decisions about what they do. They like to be free and not depend on others”
Conservation	6–36	Conformity	2	“They believe that people should do what they're told. They think people should follow rules at all times, even when no-one is watching”
		Security	2	“They believe that people should do what they're told. They think people should follow rules at all times, even when no-one is watching”
		Tradition	2	“Tradition is important to them. They try to follow the customs handed down by their religion or their family”

#### 3.3.3 Zimbardo's Time Perspective Inventory (ZTPI)

We used the Hungarian validated short version of the Zimbardo Time Perspective Inventory (ZTPI, Orosz et al., 2017; Zimbardo and Boyd, 1999), which consists of 17 items, assessing the five time perspective dimensions. Respondents rate each statement on a 5-point Likert scale, indicating how true or characteristic it is of them, with 1 representing “very untrue/uncharacteristic” and 5 indicating “very true/characteristic.” For each subscale, Table 3 displays the minimum and maximum attainable scores, the number of items, and example items. Higher scores indicated stronger specific time perspective in all cases.

**Table 3:** Time perspective dimensions of Zimbardo's Time Perspective Inventory (Zimbardo and Boyd, 1999; ZTPI, Orosz et al., 2017).

Time perspective	Theoretically achievable min.-max. scores	No. of items	Item example
Past-positive	3–15	3	“I get nostalgic about my childhood”
Past-negative	4–20	4	“I've taken my share of abuse and rejection in the past”
Present-hedonistic	3–15	3	“I take risks to put excitement in my life”
Present-fatalistic	3–15	3	“My life path is controlled by forces I cannot influence”
Future	4–20	4	“I meet my obligations to friends and authorities on time”

### 3.4 Statistical analyses

Based on earlier studies (Osgood et al., 1957; Dúll and Urbán, 1997), Principal Component Analyses (PCA) with Varimax rotation and, based on the results, Promax rotation were performed on the responses to the SDBH items to uncover the meaning dimensions. The following criteria were applied to retain items for the final factor model: (i) each item had to have a factor loading of at least 0.40 on the primary factor, and either (ii) cross-loadings on the interpreted components did not exceed 0.30, or (iii) the difference between the primary and secondary factor loadings exceeded 0.20.

Descriptive, reliability, and correlation analyses were executed in the case of all (sub)scales used in the path analysis. Considering the non-normal distribution of the variables and the aim of controlling for gender, a partial nonparametric (Spearman) correlation analysis was performed.

To explore the pattern of the variables' relationship and the possible mediation effects, a path model was prepared that included personal values and age as predictors, time perspectives as mediators, and built heritage meaning dimensions as output variables. Gender was included as a control variable, whereas other demographic characteristics were either beyond the scope of the study or showed limited variability in the present sample. Although the model was informed by previous theoretical and empirical findings, the analysis was exploratory in nature because the proposed pattern of associations has not previously been examined in the context of built heritage. Therefore, a saturated path model was specified to estimate all theoretically relevant associations and potential indirect relationships. The indirect effects involving time perspectives were examined as potential mediation patterns; however, these analyses are interpreted as statistical associations rather than evidence of causal relationships. As all variables were measured at the same time point, temporal precedence and causal direction cannot be established. The effects were estimated using 95% bias-corrected, bootstrapped confidence intervals (based on the ML estimation method) with 10,000 bootstrap replicates. The standardized regression coefficients (βs), their 95% bootstrapped confidence intervals, and the total, direct, and indirect effects were interpreted based on the STDYX output.

We used SPSS version 28.0 (IBM Corp, 2021) for PCA, descriptive and reliability analyses, JASP version 0.19.1 (JASP Team, 2024) for correlation analysis, and Mplus version 8 (Muthén and Muthén, 1998-2017) for path analysis.

## 4 Results

### 4.1 Built heritage meaning dimensions

The initial PCA with Varimax rotation (Kaiser–Meyer–Olkin measure of sampling adequacy: KMO = 0.958, Bartlett test: *p* < 0.001), identified five components with eigenvalues greater than 1 (eigenvalues: 12.166, 2.906, 1.765, 1.157, and 1.084; see Appendix Table B-1). However, the fourth and fifth components contained only a single item each without cross-loadings. Considering the Scree plot, only the first three components were retained, explaining 53.4% of the total variance. Although Varimax rotation was initially applied in accordance with the classical method proposed by Osgood et al. (1957), where connotative meaning dimensions are conceptualized as independent, the Spearman correlation coefficients between the scores of the three interpreted components (*r*_S_ = 0.632, 0.543, and 0.476) suggested that the components were not orthogonal. Therefore, an additional PCA with Promax rotation was conducted (Table 4), which also resulted in five components with eigenvalues greater than 1; however, the fourth component included two items and the fifth component one item, each showing factor loadings above 0.4 on their respective components and no meaningful cross-loadings on the first three components. As the correlation between the two items forming the fourth component was moderate (*r* = 0.442), only the first three components were retained in the Promax rotation, consistent with the initial Varimax rotation. Following the exclusion criteria, seven adjective pairs were removed from the final set, either due to high cross-loadings (two items) or because they did not load primarily on any of the identified main components (five items). The first emerging component was interpreted as *evaluation* (e.g., valuable-worthless, memorable-forgettable), the second as *human connectedness* (e.g., intimate-cold, romantic-rational), and the third as *condition* (e.g., ruined-intact, fragmented-complete).

**Table 4:** Promax-rotated component matrix of the semantic differential scale for built heritage.

No. as appeared in scale	English translation	Component				
		1	2	3	4	5
29	**thought-provoking–uninteresting**	**0.949**				
3	**special–ordinary**	**0.904**				
30	**memorable–forgettable**	**0.820**				
16	**emblematic–unremarkable**	**0.728**				
15	**exciting–boring**	**0.722**				
10	**valuable–worthless**	**0.674**				
21	**masterly–dilettante**	**0.608**				
26	**to be preserved–to be demolished**	**0.606**				
1	**fake–authentic**	**−0.556**			0.364	
2	**dignified–unrefined**	**0.515**				
18	**ornate–unadorned**		**0.850**			
6	**romantic–rational**		**0.849**			
7	**intimate–cold**		**0.824**			
17	**cold–friendly**		**−0.804**			
11	**engineered–artistic**		**−0.789**			0.405
22	**lively–deserted**		**0.676**	0.308		
13	**calming–disturbing**		**0.592**		**–**0.318	
27	**popular–unknown**		**0.554**			
14	**inviting–repulsive**		**0.545**			
24	**fragmented–complete**			**−0.904**		
5	**ruined–intact**			**−0.900**		
4	**orderly–disorderly**	0.320		**0.715**		
23	**to be renovated–renovated**		**–**0.350	**−0.553**		
20	timeless–ephemeral	0.430		0.310		
25	unimaginative–inventive	**–**0.387	**–**0.427			
8	tacky–tasteful				0.660	
12	forced–natural		−0.330		0.549	
19	detailed–simple	0.421	0.446		0.449	
9	harmonious–dissonant				**–**0.319	
28	human-scale–monumental			0.328		**–**0.832

*N* = 431.

Factor loadings are shown after Promax rotation with Kaiser normalization.

Rotation converged in 8 iterations.

Adjectives were translated into English by the first author.

Interpreted items retained for the final three-factor structure are presented in the upper part of the table in bold.

Only factor loadings above 0.30 are shown.

Exclusion criteria: (i) each item had to have a factor loading of at least 0.40 on the primary factor, and either (ii) cross-loadings on the interpreted components did not exceed 0.30, or (iii) the difference between the primary and secondary factor loadings exceeded 0.20.

### 4.2 Descriptive statistics and reliability

Table 5 presents the descriptive statistics for all variables included in subsequent analyses, along with the internal consistency of the subscales. While some Cronbach's alpha values were below 0.7, none fell into the range indicating poor reliability (below 0.6).

**Table 5:** Descriptive analysis of variables used in inferential analyses and reliability assessment of (sub)scales.

Variable	*N*	%	*M*	SD	Min	Max	Cronbach's α
Connotative meaning dimensions attributed to built heritage (mean^a^)							
Evaluation	409		55.27	6.79	23.40	70.00	0.925, 0.905, 0.900, 0.906, 0.887^b^
Human connectedness	414		41.71	4.75	31.20	56.40	0.767, 0.840, 0.848, 0.793, 0.716^b^
Condition	414		17.96	2.33	12.00	24.80	0.647, 0.734, 0.713, 0.604, 0.639^b^
Personal values							
Self-enhancement	415		15.30	4.129	4	24	0.746
Self-transcendence	416		24.06	3.559	12	30	0.657
Openness to change	415		26.51	4.950	12	36	0.759
Conservation	416		22.94	4.742	8	35	0.583
Time perspectives							
Past-positive	416		10.38	2.752	3	15	0.739
Past-negative	417		11.50	3.961	4	20	0.851
Present-hedonistic	417		8.92	2.796	3	15	0.784
Present-fatalistic	417		8.00	2.512	3	15	0.608
Future	417		14.46	3.005	4	20	0.722
Gender	431						
Male Female	154 277	35.7 64.3					
Age	431		33.61	15.32	19	84	

^a^mean of the meaning dimension scores achieved for the five heritage sites; ^b^Cronbach's alpha values calculated for the five heritage sites.

### 4.3 Correlations between SDBH, PVQ and ZTPI

The results of the partial Spearman correlation analysis are presented in Table 6. The strongest correlations (*r* = 0.475–0.718) emerged among the three built heritage meaning dimensions. *Human connectedness* and *condition* showed moderate negative correlations with self-enhancement, while all three meaning dimensions showed somewhat stronger positive correlations with self-transcendence values. Openness to change was weakly associated with *evaluation*, while all three meaning dimensions exhibited a moderate positive correlation with conservation value. *Human connectedness* and *condition* were weakly and negatively related to past-negative, while *evaluation* displayed stronger positive correlation with past-positive, and *condition* a moderate negative correlation with present hedonism time perspective. All three meaning dimensions were moderately and positively associated with future orientation and showed stronger correlations with age.

**Table 6:** Partial Spearman's correlations between built heritage meaning dimensions, value categories, and time perspective factors.

Variable	1	2	3	4	5	6	7	8	9	10	11	12	13
1. Evaluation	—												
2. Human connectedness	0.718^***^	—											
3. Condition	0.497^***^	0.475^***^	—										
4. Self-enhancement	−0.084	−0.183^***^	−0.176^***^	—									
5. Self–transcendence	0.315^***^	0.185^***^	0.172^***^	0.115^*^	—								
6. Openness to change	0.109^*^	0.032	−0.004	0.412^***^	0.285^***^	—							
7. Conservation	0.164^***^	0.099^*^	0.152^**^	0.156^**^	0.195^***^	−0.117^*^	—						
8. Past-negative	−0.091	−0.116^*^	−0.152^**^	0.175^***^	0.012	0.074	0.031	—					
9. Past-positive	0.194^***^	0.082	0.088	0.154^**^	0.141^**^	0.164^***^	0.189^***^	−0.019	—				
10. Present-hedonism	−0.029	−0.077	−0.125^*^	0.241^***^	0.073	0.574^***^	−0.241^***^	0.200^***^	0.198^***^	—			
11. Present-fatalism	0.017	0.082	0.079	−0.051	−0.088	−0.081	0.082	0.073	0.075	0.006	—		
12. Future	0.167^***^	0.104^*^	0.221^***^	−0.038	0.184^***^	−0.117^*^	0.244^***^	−0.153^**^	0.150^**^	−0.197^***^	−0.085	—	
13. Age	0.222^***^	0.314^***^	0.311^***^	−0.298^***^	−0.054	−0.222^***^	0.083	−0.217^***^	−0.120^*^	−0.337^***^	0.058	0.177^***^	—

*N* = 431; Controlled for gender; ^*^p < 0.05, ^**^p < 0.01, ^***^p < 0.001.

### 4.4 Path analysis

**Figure 2:**
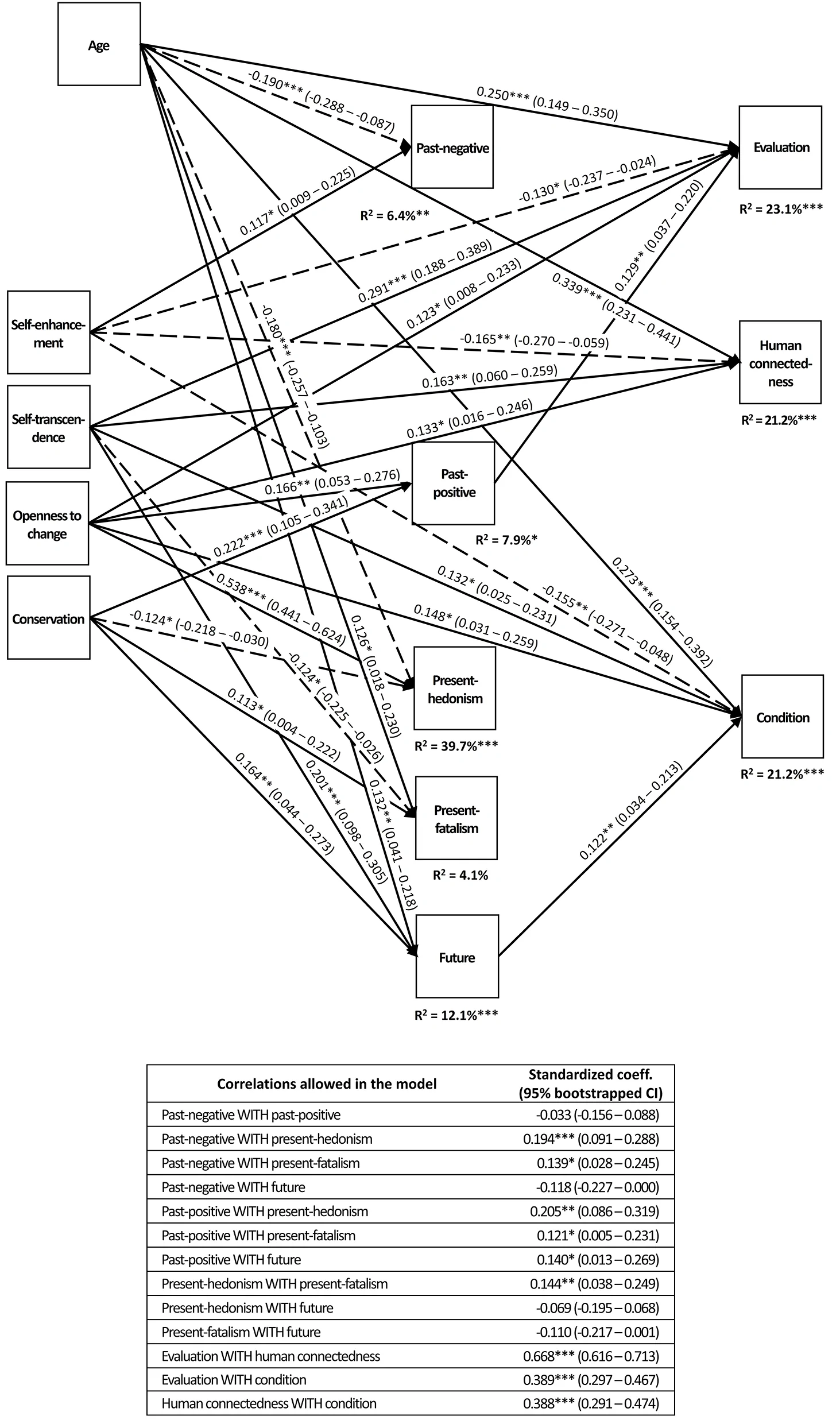
Results of the path analysis. Solid lines: positive associations; dashed lines: negative associations; for clarity, only standardized coefficients (β) with the related 95% bootstrapped confidence intervals are shown, and correlations are displayed in the table only; *p < 0.05; **p < 0.01; ***p < 0.001.

The direct associations identified in the path model are illustrated in Figure 2. The model explained 23.1% of the variance in *evaluation*, 21.2% in *human connectedness*, and 21.2% in *condition*. According to the results of the additional multiple regression analyses, no evidence of multicollinearity was detected for any of the three output variables, with VIF values ranging between 1.096–1.957, 1.095–1.958, and 1.092–1.954, respectively. The total, direct, and indirect effects found in the path analysis are summarized in Table 7 (significant results) and detailed in Appendix C (all results).

**Table 7:** Significant results of the path model revealing the associations between personal values, age, and built heritage meaning dimensions (mediators: time perspectives), controlling for gender.

Associations	Direct		Indirect: total and specific		Total	
	β (95% bootst. CI)	*p*	β (95% bootst. CI)	*p*	β (95% bootst. CI)	*p*
Self-enhancement ⇒						
⇒ Past-negative	**0.117 (0.009 to 0.225)**	**0.034**	—	—	**0.117 (0.009 to 0.225)**	**0.034**
⇒ Evaluation	**−0.130 (−0.237 to** **−0.024)**	**0.018**	0.005 (−0.018** to **0.033)	0.678	**−0.125 (−0.231 to** **−0.019)**	**0.022**
⇒ Human connectedness	**−0.165(−0.270 to** **−0.059)**	**0.002**	−0.001(−0.023 to 0.021)	0.945	**−0.166(−0.270 to** **−0.063)**	**0.002**
⇒ Condition	**−0.155 (−0.271 to** **−0.048)**	**0.006**	−0.007 (−0.036 to 0.019)	0.622	**−0.162 (−0.277 to** **−0.052)**	**0.004**
Self–transcendence ⇒						
⇒ Present–fatalism	**−0.124 (−0.225 to** **−0.026)**	**0.014**	—	—	**−0.124 (−0.225 to** **−0.026)**	**0.014**
⇒ Future	**0.201 (0.098 to 0.305)**	**<0.001**	—	—	**0.201 (0.098 to 0.305)**	**<0.001**
⇒ Evaluation	**0.291 (0.188 to 0.389)**	**<0.001**	0.020 (−0.006 to 0.055)	0.197	**0.311 (0.207 to 0.405)**	**<0.001**
⇒ Human connectedness	**0.163 (0.060 to 0.259)**	**0.001**	0.002 (−0.027 to 0.029)	0.911	**0.164 (0.064 to 0.257)**	**0.001**
⇒ Condition	**0.132 (0.025 to 0.231)**	**0.011**	0.023 (−0.004 to 0.057)	0.126	**0.155 (0.053 to 0.254)**	**0.003**
via Future			**0.025 (0.008 to 0.053)**	**0.023**		
Openness – change ⇒						
⇒ Past–positive	**0.166 (0.053 to 0.276)**	**0.004**	—	—	**0.166 (0.053 to 0.276)**	**0.004**
⇒ Present-hedonism	**0.538 (0.441 to 0.624)**	**<0.001**	—	—	**0.538 (0.441 to 0.624)**	**<0.001**
⇒ Evaluation	**0.123 (0.008 to 0.233)**	**0.031**	−0.009 (−0.081 to 0.065)	0.814	**0.114 (0.020 to 0.207)**	**0.017**
via Past-positive			***0.021 (0.005 to 0.050)***	0.056		
⇒ Human connectedness	**0.133 (0.016 to 0.246)**	**0.024**	0.002 (−0.058 to 0.062)	0.945	**0.135 (0.032 to 0.235)**	**0.010**
⇒ Condition	**0.148 (0.031 to 0.259)**	**0.011**	−0.016 (−0.085 to 0.052)	0.639	**0.131 (0.023 to 0.236)**	**0.015**
Conservation ⇒						
⇒ Past-positive	**0.222 (0.105 to 0.341)**	**<0.001**	—	—	**0.222 (0.105 to 0.341)**	**<0.001**
⇒ Present-hedonism	**−0.124 (−0.218 to** **−0.030)**	**0.010**	—	—	**−0.124 (−0.218 to** **−0.030)**	**0.010**
⇒ Present-fatalism	**0.113 (0.004 to 0.222)**	**0.044**	—	—	**0.113 (0.004 to 0.222)**	**0.044**
⇒ Future	**0.164 (0.044 to 0.273)**	**0.005**	—	—	**0.164 (0.044 to 0.273)**	**0.005**
⇒ Evaluation	0.067 (−0.043 to 0.172)	0.218	**0.040 (0.006 to 0.080)**	**0.033**	**0.106 (0.003 to 0.209)**	**0.043**
via Past-positive			**0.029 (0.008 to 0.061)**	**0.033**		
⇒ Condition via Future	0.083 (−0.021 to 0.189)	0.124	**0.043 (0.008 to 0.084)** ***0.020 (0.004 to 0.048)***	**0.026** 0.057	**0.126 (0.023 to 0.231)**	**0.017**
Age ⇒						
⇒ Past-negative	**−0.190 (−0.288 to** **−0.087)**	**<0.001**	—	—	**−0.190 (−0.288 to** **−0.087)**	**<0.001**
⇒ Present-hedonism	**−0.180 (−0.257 to** **−0.103)**	**<0.001**	—	—	**−0.180 (−0.257 to** **−0.103)**	**<0.001**
⇒ Present-fatalism	**0.126 (0.018 to 0.230)**	**0.018**	—	—	**0.126 (0.018 to 0.230)**	**0.018**
⇒ Future	**0.132 (0.041 to 0.218)**	**0.003**	—	—	**0.132 (0.041 to 0.218)**	**0.003**
⇒ Evaluation	**0.250 (0.149 to 0.350)**	**<0.001**	0.001 (−0.040 to 0.039)	0.966	**0.251 (0.159 to 0.344)**	**<0.001**
⇒ Human connectedness	**0.339 (0.231 to 0.441)**	**<0.001**	0.005 (−0.029 to 0.042)	0.777	**0.344 (0.244 to 0.443)**	**<0.001**
⇒ Condition	**0.273 (0.154 to 0.392)**	**<0.001**	0.034 (−0.002 to 0.074)	0.073	**0.308 (0.194 to 0.421)**	**<0.001**
via Future			***0.016 (0.003 to 0.039)***	0.065		
Past-positive ⇒						
⇒ Evaluation	**0.129 (0.037 to 0.220)**	**0.006**	—	—	**0.129 (0.037 to 0.220)**	**0.006**
Future ⇒						
⇒ Condition	**0.122 (0.034 to 0.213)**	**0.008**	—	—	**0.122 (0.034 to 0.213)**	**0.008**
Past-negative WITH present-hedonism	**0.194 (0.091 to 0.288)**	**<0.001**	—	—	—	—
Past-negative WITH present-fatalism	**0.139 (0.028 to 0.245)**	**0.011**	—	—	—	—
Past-positive WITH present-hedonism	**0.205 (0.086 to 0.319)**	**0.001**	—	—	—	—
Past-positive WITH present-fatalism	**0.121 (0.005 to 0.231)**	**0.036**	—	—	—	—
Past-positive WITH future	**0.140 (0.013 to 0.269)**	**0.033**	—	—	—	—
Present-hedonism WITH present-fatalism	**0.144 (0.038 to 0.249)**	**0.008**	—	—	—	—
Evaluation WITH human connectedness	**0.668 (0.616 to 0.713)**	**<0.001**	—	—	—	—
Evaluation WITH condition	**0.389 (0.297 to 0.467)**	**<0.001**	—	—	—	—
Human connectedness WITH condition	**0.388 (0.291 to 0.474)**	**<0.001**	—	—	—	—
Past-negative: **R^2^** **=** **6.4% (*****p*** **=** **0.011)** Past-positive: **R^2^** **=** **7.9% (*****p*** **=** **0.011)** Present-hedonism: **R^2^** **=** **39.7% (*****p*** **<** **0.011)** Present-fatalism: *R*^2^ = 4.1% (*p* = 0.053) Future: **R^2^** **=** **12.1% (*****p*** **<** **0.001)** Evaluation: **R^2^** **=** **23.1% (*****p*** **<** **0.001)** Human connectedness: **R^2^** **=** **21.2% (p** **<** **0.001)** Condition: **R^2^** **=** **21.2% (*****p*** **<** **0.001)**						

Bold: significant result; Bold and Italic: significant result according to the 95% bootstrapped confidence interval (despite the p-value); β: standardized coefficient; in brackets: 95% bootstrapped confidence intervals of β; R^2^: explained variance.

#### 4.4.1 Direct effects

Self-enhancement was positively associated with past-negative time perspective (TP) and negatively associated with all three built heritage meaning dimensions (BHMDs), the latter confirming H1. It showed no associations with past-positive, present-hedonism, present-fatalism, or future TPs. Self-transcendence had positive associations with future TP and all three BHMDs, the latter confirming H2, and negatively associated with present-fatalism, while showing no associations with past-negative, past-positive, or present-hedonism. Openness to change was positively associated with past-positive and present-hedonism TPs as well as all three BHMDs, but had no associations with past-negative, present-fatalism, and future TPs. Conservation was positively associated with past-positive, present-fatalism, and future TPs, and negatively associated with present-hedonism, but showed no direct associations with past-negative TP or any BHMDs. Past-positive TP was positively associated with the *evaluation* meaning dimension, partially confirming H3. Past-negative, present-hedonism, and present-fatalism TPs showed no associations with BHMDs, thus not confirming H4. Future TP was positively associated with the *condition* meaning dimension, partially confirming H5. Age was associated with higher scores on present-fatalism and future TPs, lower scores on past-negative and present-hedonism TPs and higher scores on all three BHMDs.

#### 4.4.2 Indirect effects

Self-transcendence had a positive indirect association with the *condition* meaning dimension via future TP, indicating that the total association was partially mediated. Similarly, openness to change showed a positive indirect association with *evaluation* via past-positive TP, with partial mediation of the total association. Conservation was positively associated with *evaluation* indirectly via past-positive TP and with *condition* indirectly via future TP. Given the absence of direct associations, these relationships were fully mediated, meaning that stronger conservation values were associated with stronger evaluation or condition meaning only through their links with past-positive TP or future TP, respectively. Age showed a positive indirect association with the *condition* dimension via future TP, indicating that the total association was partially mediated. Considering the exploratory nature of the research and the bootstrapping procedure used, associations with *p*-values between 0.01 and 0.05 resulting from the mediation analysis are also interpreted. In light of the multiple comparisons involved in the path analysis, these findings should be regarded cautiously as statistical trends that may provide a basis for future research.

## 5 Discussion

### 5.1 Built heritage connotative meaning dimensions

We investigated the relationship between the connotative meaning of built heritage and individual psychological orientations, focusing on personal values and time perspectives. The 30-item Semantic Differential Scale for Built Heritage, developed from the preliminary study (Appendix A), was used to reveal the main meaning dimensions of built heritage. We identified three components: *evaluation*, which refers to value judgment dichotomies also found by Osgood et al. (1957) in their original study and by other papers on the meaning of the built environment (Craik, 1968; Canter, 1969); *human connectedness*, denoting a friendly, emotional atmospheric quality, similar to invitingness (Rafiei and Gifford, 2023); and *condition*, which refers to the physical state of built heritage, similarly identified earlier as order (Franz et al., 2003), organization (Hershberger and Clements, 1969), and firmness (Marković et al., 2016). The interdependence of the factors and the high number of cross-loadings can be attributed to the shared nature of the assessed phenomena, built heritage objects, and the specificity of the adjectives provided by heritage experts. These expert-termed descriptors are often challenging for laypeople, particularly younger individuals, to distinguish clearly.

### 5.2 Explored associations between built heritage meaning dimensions, personal values, time orientations, and age

We aimed to reveal how the assessment of built heritage along connotative meaning dimensions relates to value priorities, time perspectives, and age, and whether time perspectives mediate these relationships. Personal values and time perspectives are key psychological factors influencing how people perceive, interpret, and interact with their environment (Bardi and Schwartz, 2003; Milfont and Gouveia, 2006; Clayton, 2012). Values emerged as stronger predictors of connotative meanings than time perspectives across all meaning dimensions. The presented model highlights potential pathways linking these constructs, suggesting that personal values and temporal orientations provide a complex background for built heritage assessment. All observed relationships were independent of participants' age and gender.

#### 5.2.1 Personal values

Considering the findings on personal values (PVs) addressed in RQ1, both hypotheses were fully supported. Self-enhancement values showed direct negative relationships with all built heritage meaning dimensions (BHMDs), confirming H1. The lower-order values within self-enhancement include power, achievement, and elements of hedonism. According to Schwartz (2012), the defining goal expressed by power is “social status and prestige, control or dominance over people and resources” (p. 5.), while achievement emphasizes personal success through social approval, and hedonism is defined by self-indulgence and life enjoyment. Our findings show that greater importance on these values, i.e., social superiority, ambition, wealth, and pleasure, is linked to lower connotative meaning of built heritage, including reduced value, weaker human connectedness, and lesser completeness. In other words, such values tend to co-occur with perceiving historic buildings and sites as worthless, uninteresting, cold, repulsive, fragmented, disorderly etc.

In contrast, self-transcendence value, encompassing universalism and benevolence, was directly and positively associated with all BHMDs, confirming H2. These values, i.e., concern for the welfare of others and nature, are linked to historic building meanings, such as valuable, special, friendly, calming, intact, and orderly, etc. This association aligns with the social role of built heritage that contributes to collective memory, shared identity, and community attachment, all of which are related to the meaning ascribed to historic places. Built heritage in urban settings acts as “urban reminders” that help communities remember and form a collective memory (Lewicka, 2008), catalyze the process of collective remembering and the formation of memory communities, in which people can identify with a shared past, even in the absence of direct personal experience. The primary function of collective memory is to support the maintenance and reinforcement of group identity, cohesion, and continuity (Bernáth, 2010). Considering that the association between self-transcendence and the *condition* meaning dimension was partially mediated by future TP, it can be inferred that a strong concern for others and the environment is associated with an appraisal of historic sites in terms of their physical integrity and intactness, partially due to a stronger future time orientation. This link suggests that built heritage might be conceptualized as a moral obligation, to be protected not merely for present enjoyment but as a cultural and material inheritance for posterity. Concern for the intactness of historic places suggests that these sites may be seen as vessels of continuity that connect generations, reinforcing collective memory, identity, and a sense of rootedness across time. As such, the appreciation of built heritage becomes both a reflection of prosocial values that positively affect pro-environmental behavior (De Groot and Steg, 2010; Wang et al., 2021), and a forward-looking act of cultural preservation.

As Schwartz (2012) highlights, self-enhancement and self-transcendence values are universally opposing dimensions. Our findings indicate that emphasizing either of these polarized values correlates with opposite meanings of built heritage. The BHMDs' positive relationship with self-transcendence, often identified as altruistic or biospheric values and their negative relationship with self-enhancement, regarded as egoistic values are consistent with other studies investigating associations between human values and environmental beliefs, concerns, norms and actions (De Groot and Steg, 2008; Follows and Jobber, 2000; Milfont and Gouveia, 2006; Schultz et al., 2005; Schultz, 2001).

Regarding the explorative research question on any further associations between PVs and BHMDs (RQ1), positive associations were found between openness to change and all meaning dimensions. Openness to change, located between self-transcendence and self-enhancement on Schwartz's circumplex model, comprises self-direction, stimulation, and, to some extent, hedonism values. The defining goals of these values, as suggested by Schwartz (2012), are independence of thought and action, choosing, creating, exploring, excitement, novelty, challenge, and affectively pleasant arousal. These qualities tend to co-occur with attributing higher value, stronger human connectedness, and a greater sense of completeness to built heritage. An explanation could be that built heritage represents temporal convergence, where continuity and transformation coexist. Historic sites offer a stimulating interplay between values, identity construction, permanence, and novelty, inviting the integration of past values within present contexts. Adaptive reuse and urban renewal may support identity processes by offering a platform for negotiating personal and collective narratives, especially when historic places are reinterpreted or reintegrated into contemporary cultural life based on the values and stories of communities. Thus, openness to change values are linked to an appreciation of historic buildings and sites not necessarily for their preservation *per se*, but for their potential to be re-contextualized, interpreted through critical reflection, or used as a resource for innovation and cultural discourse (Gibson and Pendlebury, 2009). These processes seem more characteristic of built heritage than of natural environments, as previous research on environmental attitudes found a significant negative correlation between openness to change values and environmental preservation (Milfont and Gouveia, 2006), and no correlation with environmental concerns in Schultz et al.'s (2005) cross-cultural studies. Further research is needed to better understand the psychological mechanisms driving these divergent associations.

#### 5.2.2 Time perspectives

Concerning TPs and BHMDs (RQ2), past-positive TP showed a positive relationship with the *evaluation* meaning dimension, partially confirming our hypothesis (H3). This suggests that pleasant, nostalgic feelings about one's past tend to co-occur with attributing greater value to built heritage. This may be because historic buildings and sites are reminders of specific collective or personal past events, while they are also enduring symbols of the past, more likely to resonate with those who associate their history with positive emotions. Future orientation was positively associated with the *condition* meaning dimension, again partially confirming our hypothesis (H5). This suggests that future orientation is associated with a more acute perception of the physical state of built heritage, particularly regarding its intactness and completeness as elements that endure into the future. By linking past, present, and future, built heritage that persists over time may support people's need for continuity and reinforces the belief that valued elements of the past will remain accessible to future generations. These findings can be paralleled with previous studies linking pro-environmental attitudes to future orientation (Ebreo and Vining, 2001; Joireman et al., 2004; Milfont and Gouveia, 2006; Pinheiro and Corral Verdugo, 2013; Carmi and Arnon, 2014; Tucholska et al., 2024), and past-positive time perspective with environmental preservation (Milfont and Gouveia, 2006). However, the hypothesis regarding the negative association between present-fatalism and BHMDs (H4) was not supported by our study. Similarly, present-hedonism and past-negative were not associated with any BHMD, answering our second research question (RQ2). These findings suggest that temporal orientations previously linked to pro-environmental attitudes may not be directly transferable to the context of built heritage connotative meanings. This may refer to the distinctive characteristics of heritage as a human-made environment that embodies historical continuity and intergenerational transmission rather than immediate personal concerns.

#### 5.2.3 The role of age

With regard to our third research question (RQ3), older respondents attributed meanings of greater value, stronger human connectedness, and more completeness to built heritage. This suggests that age is associated with more positive evaluations of historic buildings, with older people tending to perceive them as more valuable, inviting, and complete. This aligns with place attachment research, which argues that emotional bonds to physical environments strengthen over time (Rubinstein and Parmelee, 1992; Ng et al., 2005; Shamai and Ilatov, 2005; Lewicka, 2010), often explained by longer residence duration. In our study, however, age was positively related to all connotative meaning dimensions as a general affiliation with historic sites. We interpret this favorable attitude toward built heritage, which symbolically transcends human mortality, as reflecting a heightened awareness of finitude that comes with age. Historic buildings and sites may thus function as legacies or lasting testaments that connect generations and extend meaning beyond the individual lifespan. Cognitive and affective factors may also contribute: older adults are characterized by value-directed remembering and a focus on positive experiences, leading to greater life satisfaction and more positive evaluations (Castel, 2024). The transition from negativity bias in youth to a preference for positive information in later life is termed the “positivity effect” (Carstensen and DeLiema, 2018). With age, memory processes become more abstract and emotionally meaningful, shifting from detail-oriented recall to pattern recognition and semantic integration (Carstensen et al., 1999; Cabeza, 2002). Longer exposure to certain environments over the life course further deepen autobiographical associations and personal meaning (Rubinstein and Parmelee, 1992). These theoretical explanations provide possible interpretations for the observed associations, as the underlying mechanisms were not directly assessed in the present study. Nevertheless, understanding age-related differences in the connotative meaning attributed to built heritage is essential for developing heritage conservation strategies that resonate across age groups. Future research should directly examine potential mediating mechanisms such as life satisfaction, mortality salience, and emotion regulation to better explain the observed age differences.

#### 5.2.4 Mediating effects of past-positive and future time perspectives

In response to our fourth research question (RQ4), we found that specific TPs mediated the relationship between certain PVs and BHMDs. The link between self-transcendence and *condition* was partially mediated by future TP, indicating that the prioritization of the welfare of others was associated with attributing greater completeness and intactness to built heritage, with part of this association accounted for by a more forward-looking outlook. Likewise, a past-positive TP partially mediated the relationship between openness to change and *evaluation* dimension, suggesting that the valuation of novelty and challenge was associated with attributing greater value to built heritage, with this relationship partly accounted for by a more favorable orientation toward the past.

Although no direct relationships emerged between conservation values and any built heritage connotative meaning dimensions, the path analysis revealed indirect links with *evaluation* and *condition*, mediated by past-positive and future time orientations, respectively. The conservation value category, comprising security, conformity, and tradition, reflects subordination to social or cultural expectations, to present norms or enduring beliefs (Schwartz, 2012). The indirect pathway from conservation values to the meaning of greater value via a past-positive orientation suggests that valuing tradition and security is associated with a nostalgic or affectionate view of the past. Zimbardo and Boyd (1999) describe past-positive orientation as a sentimental and affirmative stance toward one's personal or cultural history. Such an orientation may be associated with stronger emotional attachment to historic built environments, which have been described as repositories of collective memory and symbols of identity (Altman and Low, 1992; Lewicka, 2008; Scannell and Gifford, 2010). Accordingly, endorsement of conservation values may co-occur with a deeper appreciation of the past, and more positive evaluations of heritage sites. Similarly, the indirect link between conservation values and *condition* meaning dimension, mediated by a future orientation, reflects a forward-looking concern for the survival of built heritage. Future-oriented individuals tend to prioritize long-term planning and the consequences of present actions (Zimbardo and Boyd, 1999). Combined with conservation-driven motivations, this orientation may be associated with greater sensitivity to the physical integrity of heritage. Through its connection with the meaning of completeness, a future-oriented outlook may also be linked to a preference for well-preserved historic buildings and sites. These relationships merit further empirical investigation.

According to our findings, older participants tended to be both more present-fatalistic and future-oriented, and less characterized by present-hedonism and a past-negative orientation. Future time perspective partially mediated the association between age and *condition* dimension, indicating that older respondents attributed greater completeness and intactness to built heritage, with part of this association linked to a more future-oriented outlook. Given that older respondents in the present study also showed higher future orientation, the observed indirect association may reflect a greater emphasis on the long-term continuity of historic places. From this perspective, well-preserved and intact heritage may be perceived as better able to fulfill its role as a cultural resource for future generations.

### 5.3 Strengths, limitations, and future research

A major contribution of the current research is its advancement of knowledge on built heritage examined from an environmental psychology perspective, with implications for urban planning and heritage management. By employing a scale specifically developed to measure the connotative meaning of built heritage and by establishing a theoretical framework linking these meaning dimensions with human values and time perspectives, the study provides a valuable contribution to environmental and behavioral research. Our findings support prior research demonstrating associations between personal values, time orientations, and pro-environmental attitudes and behavior, which until now have been primarily explored in natural environments and, to a lesser extent, the built environment. By exploring the connotative meaning of built heritage, reflecting attitudes toward it, our study extends this line of inquiry to the historic built environment. Overall, the results show that personal values and temporal orientations have significant associations with the meaning dimensions attributed to historic buildings and sites, highlighting the role of individual differences in the assessment of built heritage and its broader cultural and societal relevance.

The outcomes may inform conservation practice and policy by offering an enriched understanding of the psychological dimensions of built heritage, with relevance for urban environments and the potential to be integrated into conservation plans, international charters and conventions. They highlight the importance of systematically exploring how specific heritage sites are perceived and experienced by their stakeholders before planning conservation, restoration, or adaptive reuse interventions, thereby supporting more context-sensitive, participatory, and sustainable heritage management. The revealed associations can also inform sustainability-related communication and education in heritage conservation; however, these implications should be interpreted as theoretically informed extrapolations rather than empirically tested intervention effects. First, the mediating role of future orientation suggests that historic preservation may be understood as contributing to long-term environmental sustainability through maintenance actions, adaptive reuse, and energy-efficient retrofitting of historic buildings. This perspective may support the integration of heritage conservation within broader sustainability and circular economy agendas, both in professional practice and in environmentally conscious public communication. A second potential implication concerns the development of targeted heritage promotion and education strategies aligned with social values, including narratives that emphasize collective memory, sustainability, and intergenerational responsibility. A third implication relates to the design of age-sensitive engagement programs that highlight legacy and continuity for older adults, and creativity and adaptive reuse for younger audiences. Fourth, connotative meaning dimensions may also be considered in heritage assessments alongside architectural and historical criteria, as they provide insight into perceived public significance, potentially supporting prioritization in funding and conservation decisions. Finally, community involvement in conservation projects may benefit from approaches that explore and integrate people's values and meanings, potentially enhancing feelings of connection, belonging, and responsibility.

A particularly noteworthy finding is the emergence of *condition* as a distinct connotative meaning dimension reflecting perceived physical and material states. This suggests that the condition of built heritage is salient not only to conservation professionals but also to laypeople, forming an integral part of how historic places are perceived and interpreted. Although these perceptions are not equivalent to professional assessments of conservation status or material deterioration, they point to the potential of participatory heritage approaches, in which lay observations may contribute to the identification of visible building pathology or to complementary forms of heritage monitoring. Such approaches could help foster shared responsibility for the long-term care and sustainability of built heritage between experts and the public. Further research is needed to examine the relationship between perceived condition and objective conservation indicators, as well as to explore how public education may strengthen lay competence in recognizing and communicating conservation-related issues.

We acknowledge several limitations in the study. The first concerns the selection of visual stimuli. To facilitate the research, we consulted heritage professionals and employed visual stimuli, given the lack of consensus on a clear definition of built heritage or historic buildings and sites, and on how this broad concept is represented in people's minds. The stimuli comprised of panoramic photos of five built heritage sites to accommodate participants' attention spans and survey completion time. These were intentionally chosen to represent diverse heritage types as the aim was to explore general patterns of connotative meaning associated with built heritage. Since they differed in architectural style, function, material, scale, and visual features, the extracted connotative meaning dimensions may partly reflect the characteristics of the selected sites and should be interpreted within the context of the presented heritage examples. Future studies could examine a broader and more systematically varied set of heritage sites to further investigate whether the identified meaning dimensions generalize across different types of built heritage environments. Second, although respondents were encouraged to engage deeply with the images, their assessments may not fully reflect real-life experiences. While the use of 360° panoramic images is widely used and supported in environmental psychology research as a valid method for simulating real-world settings (Stamps, 1990, 2010), we acknowledge that different modes of presentation (e.g., immersive virtual environments) may elicit different affective responses, and that such stimuli cannot fully reproduce embodied, *in-situ* experiences of place. Furthermore, participants' prior personal experience with the depicted sites was not assessed; therefore, familiarity or autobiographical associations may have influenced the observed meaning dimensions. Third, the SDBH should be interpreted as a context-dependent and exploratory semantic differential tool rather than a fully invariant psychometric measurement instrument. Accordingly, the identified connotative meaning structure reflects a fluid configuration that may vary across samples and heritage contexts. While PCA was used to derive meaning dimensions, exploratory and hypothesis-driven analyses were conducted within the same dataset, which limits the separation between exploratory and confirmatory procedures and may raise concerns regarding robustness and potential overfitting. Also, reliance on convenience sampling resulted in a predominantly young cohort, which may have contributed to the emergence of a simpler, three-factor structure of meaning dimensions that differed from the preliminary study findings. A more diverse, representative sample could yield more complex and generalizable results. Future research should test the stability of these dimensions across independent samples and settings. Fourth, given the cross-sectional design, associations in the path model do not imply causal or unidirectional relationships. Although the proposed path model was informed by theoretical considerations regarding the relationships among values, time perspectives, and built heritage meanings, all variables were measured concurrently, and we examined patterns of associations rather than causal relationships. Further studies are needed to examine the directionality and potential causal mechanisms underlying these associations. Fifth, the generalizability of the findings is limited, as the research involved primarily Hungarian participants, mostly students. Prior research indicates cross-cultural differences in value priorities (Schwartz, 1994) and time perspectives (Stolarski et al., 2015). Sixth, although the study employed established and previously validated measures of personal values (Schwartz, 2012) and time perspective (Orosz et al., 2017), internal consistency may vary across samples and research contexts. Some of the scales in the present study showed lower reliability than is typically recommended. Because measurement unreliability introduces random measurement error, the estimated associations in the path analysis may be attenuated (Cole and Preacher, 2014; Fritz et al., 2016). Consequently, the absence of significant effects should be interpreted with caution. Further research using alternative measures and longitudinal designs is needed to determine whether the findings (including null findings) reflect the specific psychological nature of built heritage connotative meaning or measurement-related limitations. Finally, the study was not preregistered due to its predominantly exploratory design. As a result, the possibility of inflated Type I error associated with multiple testing and data-driven model refinement cannot be fully excluded. Future research should aim to preregister hypotheses and analytical plans to further strengthen the validity and reproducibility of findings.

While studies examining how people attribute meaning to historic buildings remain limited, understanding the underlying perceptions is central to informing preservation strategies and enhancing engagement with heritage sites. Building on our findings, future research could explore the factors driving the revealed associations, particularly those without prior literature, such as the links with openness to change and conservation values. To enhance ecological validity, studies could be conducted on-site or use advanced technologies, such as computer simulations or virtual reality, to create immersive experiences. More diverse sampling across broader age ranges would improve the robustness and generalizability of the results. Additional individual factors, including cultural background and prior experience, could be examined for their influence on connotative meaning. Finally, expanding the focus to other types of built heritage, including modernist buildings, dissonant heritage, or contemporary constructions in historic styles, would provide a more comprehensive understanding of people's assessment of built heritage.

## 6 Conclusions

While time perspectives and personal values have long been recognized as important constructs in environmental psychology, their combined relationship with the connotative meaning attributed to the built historic environment has not been previously examined. To address this gap, the present study applied a semantic differential scale for built heritage, developed by our research team (Fogarasi et al., 2023), alongside Zimbardo and Boyd (1999) ZTPI time perspectives, and Schwartz et al.'s (2001) PVQ value categories. We developed and validated a scale to measure people's connotative meaning of built heritage, identifying three dimensions—*evaluation, human connectedness*, and *condition*—and revealed associations with personal values, time perspectives, and age. Self-transcendence and openness to change were positively associated with all meaning dimensions, with future and past-positive time orientations partially mediating links to *condition* and *evaluation*, respectively. Self-enhancement values showed direct negative associations with all dimensions, while conservation values were linked to *evaluation* and *condition* only indirectly through past-positive and future temporal perspectives, respectively. Older respondents attributed greater value, connectedness, and completeness to the built historic environment. Overall, these findings extend environmental and urban place meaning research (Namaz and Tvergyák, 2023) by demonstrating that value and temporal orientations are associated with the perceptions of the historic built environment.

Recognizing these individual differences can inform conservation strategies and policy by incorporating psychological factors alongside traditional evaluations. Although built heritage preservation is not often at the forefront of societal concerns, public engagement is pivotal to the survival of historic buildings and sites in both urban and other contexts. Understanding how people perceive and attribute meaning to built heritage is fundamental for effective conservation, community involvement, and sustaining a historic environment that reinforces identity and wellbeing in a rapidly changing world. This research contributes to a transdisciplinary approach by integrating psychological evidence with heritage studies, illuminating the factors that influence the connotative meaning of built heritage and highlighting its significance for both urban and rural environments, where historic buildings play a central role in shaping social life, local identity, and the experience of place.

## Data Availability

The anonymized dataset supporting the conclusions of this article, along with the analysis code, will be made available in a university institutional repository upon publication. Access to the dataset and code will be provided upon reasonable request and in accordance with applicable ethical and data protection regulations.
