# Supplementary material for: Beyond stone and timber: the connotative meaning of built heritage, its associations with personal values and the mediating role of time orientations
Source: Front Psychol. 2026 Aug 26;17:1903386. doi: 10.3389/fpsyg.2026.1903386 (PMC13553351; doi:10.3389/fpsyg.2026.1903386)
Supplement: Supplementary file 1 [file Supplementary_file_1.docx]

**Appendix A. Preliminary Study**

# 1. Testing the semantic differential scale developed for built heritage

## 1.1 Design and participants

The 40-item semantic differential scale developed for built heritage (Fogarasi et al., 2023) was tested online in Hungarian between January and October 2021 using Qualtrics. The survey was distributed via social media and integrated into a university course, where undergraduate students earned credit for completing the questionnaire and encouraging family and friends to participate. The scale was administered to two samples: heritage professionals and non-expert laypeople. For the purposes of the current study, we report on the laypeople sample, which comprised 213 participants (66% women, M_age_ = 52.0 years, SD_age_ = 14.0, min: 21 years, max: 88 years).

The semantic differential scale uses pairs of opposing adjectives to describe the subject, rated by participants on a seven-point bipolar scale. Given the unique qualities within the built environment, a scale specifically designed for built heritage is likely to yield more accurate and meaningful results than one based on generic terms. The scale was developed using a rigorous methodology grounded in previous research (Dúll & Urbán, 1997; Osgood et al., 1957). A key feature of its design (Fogarasi et al., 2023; Fogarasi & Dúll, 2021) was the continuous involvement of heritage professionals—experts with in-depth knowledge of the subject— at each stage. This approach served two purposes: to ensure the adjectives accurately described the subject, and to mirror the real-world context in which laypeople encounter professional terminology. Initially, experts were consulted to identify examples of built heritage, supported by visual illustrations where possible, yielding 368 responses from 21 participants. A group of three heritage experts selected the 25 most representative examples, for which professionals then contributed adjectives, producing 1205 responses from 22 respondents. Three researchers, a psychologist, a heritage professional, and a non-expert in either field, collaboratively coded the adjectives, reaching consensus on the 40 most prominent and frequently occurring responses. Finally, heritage professionals proposed corresponding antonyms, resulting in the finalized 40-item bipolar scale.

Participants first answered basic demographic questions, including age, gender, education, profession, place of residence. The study used 360° panoramic photos of five heritage sites, selected from the 25 images used in the preliminary study based on their prominence in responses and representing the most typical types of built heritage: ruins (church or castle), historic urban squares, vernacular architecture, symbolic monuments, and industrial heritage. The five panoramic photos can be viewed via the following links: [Zsámbék ruin church](https://www.google.hu/maps/@47.5495523,18.7147241,3a,75y,88.75h,104.82t/data=!3m8!1e1!3m6!1sAF1QipPLAw0GrvqDh7r-E2lmuNrE36CGmhLkPzTDe1vM!2e10!3e11!6shttps:%2F%2Flh3.googleusercontent.com%2Fp%2FAF1QipPLAw0GrvqDh7r-E2lmuNrE36CGmhLkPzTDe1vM%3Dw900-h600-k-no-pi-14.824067946842419-ya15.421031033657187-ro0-fo100!7i8298!8i4149?entry=ttu&g_ep=EgoyMDI1MDkzMC4wIKXMDSoASAFQAw%3D%3D), [Sopron town square](https://www.google.hu/maps/@47.686367,16.591145,3a,75y,299.94h,99.7t/data=!3m8!1e1!3m6!1sAF1QipOqFwZnjVego_YwSOG36U9SkfzPCDCqZSPrpVSo!2e10!3e11!6shttps:%2F%2Flh3.googleusercontent.com%2Fp%2FAF1QipOqFwZnjVego_YwSOG36U9SkfzPCDCqZSPrpVSo%3Dw900-h600-k-no-pi-9.700000000000003-ya320.94-ro0-fo100!7i6656!8i3328?entry=ttu&g_ep=EgoyMDI1MDkzMC4wIKXMDSoASAFQAw%3D%3D), [Füzér village house](https://www.google.hu/maps/@48.5419435,21.455453,3a,75y,355.32h,75t/data=!3m8!1e1!3m6!1sAF1QipPs9QgPbTdWpmhr4A2OBfHRfkVtmQ2YL3emAf3D!2e10!3e11!6shttps:%2F%2Flh3.googleusercontent.com%2Fp%2FAF1QipPs9QgPbTdWpmhr4A2OBfHRfkVtmQ2YL3emAf3D%3Dw900-h600-k-no-pi15-ya40.87554199218749-ro0-fo100!7i8192!8i4096?entry=ttu&g_ep=EgoyMDI1MDkzMC4wIKXMDSoASAFQAw%3D%3D), [Chain Bridge and Danube panorama](https://www.google.hu/maps/@47.4987105,19.042094,3a,75y,236.64h,73.14t/data=!3m8!1e1!3m6!1sAF1QipOMPkd0wZfsm6c4gDwfdXzLuuv5u1eXI30Gr9U!2e10!3e11!6shttps:%2F%2Flh5.googleusercontent.com%2Fp%2FAF1QipOMPkd0wZfsm6c4gDwfdXzLuuv5u1eXI30Gr9U%3Dw900-h600-k-no-pi16.86-ya236.64-ro0-fo100!7i10240!8i5120?entry=ttu&g_ep=EgoyMDI1MDkzMC4wIKXMDSoASAFQAw%3D%3D), [Kelenföld Power Plant](https://www.google.hu/maps/@47.4620056,19.0568326,3a,75y,226.98h,86.08t/data=!3m8!1e1!3m6!1sAF1QipNjRFEriTl8hQAn-LymUgbJlzSIcidlF_kTY8ve!2e10!3e11!6shttps:%2F%2Flh3.googleusercontent.com%2Fp%2FAF1QipNjRFEriTl8hQAn-LymUgbJlzSIcidlF_kTY8ve%3Dw900-h600-k-no-pi3.9200000000000017-ya166.17999694824218-ro0-fo100!7i7680!8i3840?entry=ttu&g_ep=EgoyMDI1MDkzMC4wIKXMDSoASAFQAw%3D%3D). The all-round panoramic format enhances ecological validity by providing a more realistic representation of the environment than single-perspective images. It is also more time-efficient than videos while allowing participants to interact with the space, freely "move” within it, and explore the surroundings. Respondents were shown the five photos in random order, with instructions before each image to imagine themselves in the space, spend time exploring details, and immerse themselves in the environment before responding. After each image, they completed the scale, rating items on a 7-point Likert scale anchored by opposing adjectives (e.g., “fake–authentic”). Ethical approval was obtained from the Research Ethics Committee of the Faculty of Education and Psychology, ELTE Eötvös Loránd University, Budapest University, No. 2021/282-1 (May 27, 2021). Participants agreed to a written consent.

## 1.2 Results

The resulting dataset contained ratings for each heritage site across all 40 adjective pairs. As not all photos were assessed by every participant, the database comprised 960 rows, corresponding to the total number of ratings for each adjective pair. Following established methodology (Dúll & Urbán, 1997; Osgood et al., 1957), Principal Component Analysis (PCA) with Varimax Rotation was performed using SPSS version 28.0 (IBM Corp., 2021). The PCA yielded four components, explaining a total variance of 56.3%. The meaning dimensions were identified as (1) *evaluation* (containing adjective pairs such as valuable-worthless and memorable-forgettable) representing value judgment dichotomies, in line with Osgood’s findings; (2) *complexity* (including pairs like detailed-simple and ornate-unadorned) reflecting both physical attributes, e.g., ornamentation and intricate details, and abstract qualities, such as continuity, timelessness, and the manifestation of social and historical interconnectedness that embody the less consciously perceived meaning of heritage environments; (3) *human connectedness* (comprising pairs like intimate-cold and inviting-repulsive) capturing perceptual qualities of historic buildings and sites that emphasize a sense of personal connection and relatedness, fundamental to person-environment relationships; and (4) *condition* (including pairs such as ruined-intact and fragmented-complete) indicating the physical state, level of deterioration, and orderliness of heritage sites. A high number of correlations, mostly within the 21-item *evaluation* factor, and some adjective pairs not loading on any component, led to the elimination of several items. This process resulted in a reduced 30-item scale for laypeople, which was used in the subsequent study.

**Appendix B. Results of the PCA with Varimax rotation**

**Table B.1**

| Varimax-rotated component matrix of the semantic differential scale for built heritage. | | | | | | |
| --- | --- | --- | --- | --- | --- | --- |
| No. as appeared in scale | English translation | Component | | | | |
|  |  | 1 | 2 | 3 | 4 | 5 |
| 30 | **memorable - forgettable** | **0.783** |  |  |  |  |
| 3 | **special - ordinary** | **0.779** |  |  |  |  |
| 29 | **thought-provoking - uninteresting** | **0.758** |  |  |  |  |
| 16 | **emblematic - unremarkable** | **0.720** | 0.312 |  |  |  |
| 15 | **exciting - boring** | **0.707** |  |  |  |  |
| 10 | **valuable - worthless** | **0.701** | 0.311 |  |  |  |
| 21 | **masterly - dilettante** | **0.666** |  |  |  | 0.335 |
| 1 | **fake - authentic** | **-0.514** |  |  | 0.393 |  |
| 7 | **intimate - cold** |  | **0.768** |  |  |  |
| 17 | **cold - friendly** |  | **-0.759** |  |  |  |
| 18 | **ornate - unadorned** |  | **0.755** |  |  |  |
| 6 | **romantic - rational** | 0.310 | **0.745** |  |  |  |
| 13 | **calming - disturbing** |  | **0.617** |  | -0.379 |  |
| 11 | **engineered - artistic** |  | **-0.599** |  |  | 0.424 |
| 5 | **ruined - intact** |  |  | **-0.840** |  |  |
| 24 | **fragmented - complete** |  |  | **-0.834** |  |  |
| 4 | **orderly - disorderly** | 0.331 |  | **0.654** |  |  |
| 26 | to be preserved - to be demolished | 0.656 | 0.406 |  |  |  |
| 2 | dignified - unrefined | 0.616 | 0.378 |  |  |  |
| 25 | unimaginative - inventive | -0.512 | -0.497 |  |  |  |
| 20 | timeless - ephemeral | 0.505 |  | 0.366 |  |  |
| 22 | lively - deserted |  | 0.654 | 0.443 |  |  |
| 14 | inviting - repulsive | 0.504 | 0.607 |  | -0.311 |  |
| 27 | popular - unknown | 0.430 | 0.580 |  |  |  |
| 19 | detailed - simple | 0.460 | 0.473 |  | 0.326 |  |
| 9 | harmonious - dissonant | 0.391 | 0.401 | 0.363 | -0.368 |  |
| 23 | to be renovated - renovated |  | -0.362 | -0.563 |  |  |
| 8 | tacky - tasteful |  |  |  | 0.646 |  |
| 12 | forced - natural | -0.306 | -0.402 |  | 0.585 |  |
| 28 | human-scale - monumental |  |  |  |  | -0.763 |
| *Note:* N=431. Factor loadings are shown after Varimax rotation with Kaiser normalization. Rotation converged in 8 iterations. Adjectives were translated into English by the first author. Only factor loadings above 0.30 are shown. Interpreted items retained for the final three-factor structure are presented in the upper part of the table in bold. Exclusion criteria: (i) each item had to have a factor loading of at least 0.40 on the primary factor, and (ii) cross-loadings on the interpreted components did not exceed 0.30, or (iii) the difference between the primary and secondary factor loadings exceeded 0.20. | | | | | | |

**Appendix C.	Results of the path analysis revealing the associations between personal values, age, and built heritage meaning dimensions**

| **Table C.1**  Results of the path model revealing the associations between personal values, age, and built heritage meaning dimensions | | | | | | |
| --- | --- | --- | --- | --- | --- | --- |
| **Associations** | **Direct** | | **Indirect:**  **total and specific** | | **Total** | |
|  | β (95% bootst. CI) | p | β (95% bootst. CI) | p | β (95% bootst. CI) | p |
| **Self-enhancement ⇨** |  |  |  |  |  |  |
| ⇨ Past-negative | **0.117 (0.009 – 0.225)** | **0.034** | — | — | **0.117 (0.009 – 0.225)** | **0.034** |
| ⇨ Past-positive | 0.020 (-0.095 – 0.142) | 0.737 | — | — | 0.020 (-0.095 – 0.142) | 0.737 |
| ⇨ Present-hedonism | -0.011 (-0.109 – 0.089) | 0.833 | — | — | -0.011 (-0.109 – 0.089) | 0.833 |
| ⇨ Present-fatalism | -0.031 (-0.145 – 0.086) | 0.606 | — | — | -0.031 (-0.145 – 0.086) | 0.606 |
| ⇨ Future | 0.000 (-0.108 – 0.104) | 0.997 | — | — | 0.000 (-0.108 – 0.104) | 0.997 |
| ⇨ Evaluation  via Past-negative  via Past-positive  via Present-hedonism  via Present-fatalism  via Future | **-0.130 (-0.237 – -0.024)** | **0.018** | 0.005 (-0.018 – 0.033)  0.001 (-0.010 – 0-018)  0.003 (-0.013 – 0.022)  0.000 (-0.005 – 0.014)  0.001 (-0.003 – 0.015)  0.000 (-0.009 – 0.009) | 0.678  0.866  0.755  0.910  0.779  0.998 | **-0.125 (-0.231 – -0.019)** | **0.022** |
| ⇨ Human connectedness  via Past-negative  via Past-positive  via Present-hedonism  via Present-fatalism  via Future | **-0.165 (-0.270 – -0.059)** | **0.002** | -0.001 (-0.023 – 0.021)  -0.002 (-0.019 – 0.010)  0.002 (-0.007 – 0.019)  0.000 (-0.004 – 0.009)  0.000 (-0.013 – 0.004)  0.000 (-0.005 – 0.006) | 0.945  0.763  0.784  0.944  0.891  0.999 | **-0.166 (-0.270 – -0.063)** | **0.002** |
| ⇨ Condition  via Past-negative  via Past-positive  via Present-hedonism  via Present-fatalism  via Future | **-0.155 (-0.271 – -0.048)** | **0.006** | -0.007 (-0.036 – 0.019)  -0.008 (-0.030 – 0.002)  0.002 (-0.007 – 0.018)  0.000 (-0.005 – 0.011)  -0.001 (-0.015 – 0.003)  0.000 (-0.015 – 0.014) | 0.622  0.316  0.776  0.914  0.777  0.997 | **-0.162 (-0.277 – -0.052)** | **0.004** |
| **Self-transcendence ⇨** |  |  |  |  |  |  |
| ⇨ Past-negative | 0.002 (-0.101 – 0.099) | 0.963 | — | — | 0.002 (-0.101 – 0.099) | 0.963 |
| ⇨ Past-positive | 0.010 (-0.096 – 0.119) | 0.849 | — | — | 0.010 (-0.096 – 0.119) | 0.849 |
| ⇨ Present-hedonism | -0.066 (-0.155 – 0.015) | 0.122 | — | — | -0.066 (-0.155 – 0.015) | 0.122 |
| ⇨ Present-fatalism | **-0.124 (-0.225 – -0.026)** | **0.014** | — | — | **-0.124 (-0.225 – -0.026)** | **0.014** |
| ⇨ Future | **0.201 (0.098 – 0.305)** | **<0.001** | — | — | **0.201 (0.098 – 0.305)** | **<0.001** |
| ⇨ Evaluation  via Past-negative  via Past-positive  via Present-hedonism  via Present-fatalism  via Future | **0.291 (0.188 – 0.389)** | **<0.001** | 0.020 (-0.006 – 0.055)  0.000 (-0.005 – 0.006)  0.001 (-0.012 – 0.018)  0.003 (-0.004 – 0.019)  0.004 (-0.005 – 0.022)  0.011 (-0.008 – 0.037) | 0.197  0.993  0.859  0.590  0.517  0.330 | **0.311 (0.207 – 0.405)** | **<0.001** |
| ⇨ Human connectedness  via Past-negative  via Past-positive  via Present-hedonism  via Present-fatalism  via Future | **0.163 (0.060 – 0.259)** | **0.001** | 0.002 (-0.027 – 0.029)  0.000 (-0.007 – 0.006)  0.001 (-0.008 – 0.015)  0.001 (-0.005 – 0.014)  -0.002 (-0.018 – 0.011)  0.001 (-0.019 – 0.021) | 0.911  0.988  0.877  0.758  0.769  0.884 | **0.164 (0.064 – 0.257)** | **0.001** |
| ⇨ Condition  via Past-negative  via Past-positive  via Present-hedonism  via Present-fatalism  via Future | **0.132 (0.025 – 0.231)** | **0.011** | 0.023 (-0.004 – 0.057)  0.000 (-0.011 – 0.008)  0.001 (-0.008 – 0.015)  0.002 (-0.004 – 0.018)  -0.004 (-0.021 – 0.005)  **0.025 (0.008 – 0.053)** | 0.126  0.971  0.873  0.625  0.499  **0.023** | **0.155 (0.053 – 0.254)** | **0.003** |
| **Openness to change ⇨** |  |  |  |  |  |  |
| ⇨ Past-negative | -0.012 (-0.123 – 0.102) | 0.840 | — | — | -0.012 (-0.123 – 0.102) | 0.840 |
| ⇨ Past-positive | **0.166 (0.053 – 0.276)** | **0.004** | — | — | **0.166 (0.053 – 0.276)** | **0.004** |
| ⇨ Present-hedonism | **0.538 (0.441 – 0.624)** | **<0.001** | — | — | **0.538 (0.441 – 0.624)** | **<0.001** |
| ⇨ Present-fatalism | 0.030 (-0.090 – 0.152) | 0.622 | — | — | 0.030 (-0.090 – 0.152) | 0.622 |
| ⇨ Future | -0.104 (-0.220 – 0.012) | 0.081 | — | — | -0.104 (-0.220 – 0.012) | 0.081 |
| ⇨ Evaluation  via Past-negative  via Past-positive  via Present-hedonism  via Present-fatalism  via Future | **0.123 (0.008 – 0.233)** | **0.031** | -0.009 (-0.081 – 0.065)  0.000 (-0.008 – 0.005)  ***0.021 (0.005 – 0.050)***  -0.023 (-0.095 – 0.046)  -0.001 (-0.015 – 0.003)  -0.006 (-0.030 – 0.003) | 0.814  0.970  0.056  0.516  0.781  0.446 | **0.114 (0.020 – 0.207)** | **0.017** |
| ⇨ Human connectedness  via Past-negative  via Past-positive  via Present-hedonism  via Present-fatalism  via Future | **0.133 (0.016 – 0.246)** | **0.024** | 0.002 (-0.058 – 0.062)  0.000 (-0.005 – 0.009)  0.013 (-0.002 – 0.039)  -0.011 (-0.071 – 0.048)  0.000 (-0.004 – 0.013)  -0.001 (-0.016 – 0.009) | 0.945  0.949  0.190  0.716  0.894  0.897 | **0.135 (0.032 – 0.235)** | **0.010** |
| ⇨ Condition  via Past-negative  via Past-positive  via Present-hedonism  via Present-fatalism  via Future | **0.148 (0.031 – 0.259)** | **0.011** | -0.016 (-0.085 – 0.052)  0.001 (-0.007 – 0.015)  0.013 (-0.001 – 0.041)  -0.019 (-0.084 – 0.042)  0.001 (-0.003 – 0.015)  -0.013 (-0.037 – 0.000) | 0.639  0.876  0.186  0.555  0.788  0.157 | **0.131 (0.023 – 0.236)** | **0.015** |
| **Conservation ⇨** |  |  |  |  |  |  |
| ⇨ Past-negative | 0.047 (-0.065 – 0.160) | 0.418 | — | — | 0.047 (-0.065 – 0.160) | 0.418 |
| ⇨ Past-positive | **0.222 (0.105 – 0.341)** | **<0.001** | — | — | **0.222 (0.105 – 0.341)** | **<0.001** |
| ⇨ Present-hedonism | **-0.124 (-0.218 – -0.030)** | **0.010** | — | — | **-0.124 (-0.218 – -0.030)** | **0.010** |
| ⇨ Present-fatalism | **0.113 (0.004 – 0.222)** | **0.044** | — | — | **0.113 (0.004 – 0.222)** | **0.044** |
| ⇨ Future | **0.164 (0.044 – 0.273)** | **0.005** | — | — | **0.164 (0.044 – 0.273)** | **0.005** |
| ⇨ Evaluation  via Past-negative  via Past-positive  via Present-hedonism  via Present-fatalism  via Future | 0.067 (-0.043 – 0.172) | 0.218 | **0.040 (0.006 – 0.080)**  0.000 (-0.005 – 0.012)  **0.029 (0.008 – 0.061)**  0.005 (-0.009 – 0.028)  -0.004 (-0.021 – 0.005)  0.009 (-0.007 – 0.033) | **0.033**  0.906  **0.033**  0.551  0.527  0.352 | **0.106 (0.003 – 0.209)** | **0.043** |
| ⇨ Human connectedness  via Past-negative  via Past-positive  via Present-hedonism  via Present-fatalism  via Future | 0.056 (-0.047 – 0.156) | 0.279 | 0.022 (-0.011 – 0.058)  -0.001 (-0.015 – 0.004)  0.018 (-0.003 – 0.049)  0.003 (-0.011 – 0.019)  0.002 (-0.009 – 0.021)  0.001 (-0.015 – 0.020) | 0.208  0.843  0.175  0.735  0.783  0.890 | 0.078 (-0.020 – 0.176) | 0.122 |
| ⇨ Condition  via Past-negative  via Past-positive  via Present-hedonism  via Present-fatalism  via Future | 0.083 (-0.021 – 0.189) | 0.124 | **0.043 (0.008 – 0.084)**  -0.003 (-0.022 – 0.003)  0.018 (-0.001 – 0.050)  0.004 (-0.009 – 0.026)  0.004 (-0.004 – 0.023)  ***0.020 (0.004 – 0.048)*** | **0.026**  0.576  0.163  0.599  0.535  0.057 | **0.126 (0.023 – 0.231)** | **0.017** |
| **Age ⇨** |  |  |  |  |  |  |
| ⇨ Past-negative | **-0.190 (-0.288 – -0.087)** | **<0.001** | — | — | **-0.190 (-0.288 – -0.087)** | **<0.001** |
| ⇨ Past-positive | -0.063 (-0.162 – 0.042) | 0.228 | — | — | -0.063 (-0.162 – 0.042) | 0.228 |
| ⇨ Present-hedonism | **-0.180 (-0.257 – -0.103)** | **<0.001** | — | — | **-0.180 (-0.257 – -0.103)** | **<0.001** |
| ⇨ Present-fatalism | **0.126 (0.018 – 0.230)** | **0.018** | — | — | **0.126 (0.018 – 0.230)** | **0.018** |
| ⇨ Future | **0.132 (0.041 – 0.218)** | **0.003** | — | — | **0.132 (0.041 – 0.218)** | **0.003** |
| ⇨ Evaluation  via Past-negative  via Past-positive  via Present-hedonism  via Present-fatalism  via Future | **0.250 (0.149 – 0.350)** | **<0.001** | 0.001 (-0.040 – 0.039)  -0.002 (-0.022 – 0.018)  -0.008 (-0.028 – 0.004)  0.008 (-0.015 – 0.034)  -0.004 (-0.021 – 0.006)  0.007 (-0.005 – 0.027) | 0.966  0.857  0.306  0.530  0.507  0.367 | **0.251 (0.159 – 0.344)** | **<0.001** |
| ⇨ Human connectedness  via Past-negative  via Past-positive  via Present-hedonism  via Present-fatalism  via Future | **0.339 (0.231 – 0.441)** | **<0.001** | 0.005 (-0.029 – 0.042)  0.003 (-0.016 – 0.027)  -0.005 (-0.024 – 0.002)  0.004 (-0.017 – 0.025)  0.002 (-0.011 – 0.020)  0.001 (-0.012 – 0.015) | 0.777  0.752  0.427  0.722  0.775  0.888 | **0.344 (0.244 – 0.443)** | **<0.001** |
| ⇨ Condition  via Past-negative  via Past-positive  via Present-hedonism  via Present-fatalism  via Future | **0.273 (0.154 – 0.392)** | **<0.001** | 0.034 (-0.002 – 0.074)  0.013 (-0.005 – 0.040)  -0.005 (-0.023 – 0.002)  0.006 (-0.014 – 0.032)  0.004 (-0.005 – 0.023)  ***0.016 (0.003 – 0.039)*** | 0.073  0.260  0.394  0.572  0.517  0.065 | **0.308 (0.194 – 0.421)** | **<0.001** |
| **Past-negative ⇨** |  |  |  |  |  |  |
| ⇨ Evaluation | 0.009 (-0.086 – 0.105) | 0.851 | — | — | 0.009 (-0.086 – 0.105) | 0.851 |
| ⇨ Human connectedness | -0.018 (-0.123 – 0.086) | 0.738 | — | — | -0.018 (-0.123 – 0.086) | 0.738 |
| ⇨ Condition | -0.067 (-0.168 – 0.036) | 0.204 | — | — | -0.067 (-0.168 – 0.036) | 0.204 |
| **Past-positive ⇨** |  |  |  |  |  |  |
| ⇨ Evaluation | **0.129 (0.037 – 0.220)** | **0.006** | — | — | **0.129 (0.037 – 0.220)** | **0.006** |
| ⇨ Human connectedness | 0.079 (-0.024 – 0.178) | 0.121 | — | — | 0.079 (-0.024 – 0.178) | 0.121 |
| ⇨ Condition | 0.081 (-0.014 – 0.176) | 0.095 | — | — | 0.081 (-0.014 – 0.176) | 0.095 |
| **Present-hedonism ⇨** |  |  |  |  |  |  |
| ⇨ Evaluation | -0.043 (-0.173 – 0.086) | 0.512 | — | — | -0.043 (-0.173 – 0.086) | 0.512 |
| ⇨ Human connectedness | -0.020 (-0.128 – 0.091) | 0.713 | — | — | -0.020 (-0.128 – 0.091) | 0.713 |
| ⇨ Condition | -0.035 (-0.152 – 0.079) | 0.552 | — | — | -0.035 (-0.152 – 0.079) | 0.552 |
| **Present-fatalism ⇨** |  |  |  |  |  |  |
| ⇨ Evaluation | -0.034 (-0.126 – 0.058) | 0.462 | — | — | -0.034 (-0.126 – 0.058) | 0.462 |
| ⇨ Human connectedness | 0.016 (-0.086 – 0.120) | 0.755 | — | — | 0.016 (-0.086 – 0.120) | 0.755 |
| ⇨ Condition | 0.034 (-0.055 – 0.126) | 0.459 | — | — | 0.034 (-0.055 – 0.126) | 0.459 |
| **Future ⇨** |  |  |  |  |  |  |
| ⇨ Evaluation | 0.055 (-0.053 – 0.157) | 0.310 | — | — | 0.055 (-0.053 – 0.157) | 0.310 |
| ⇨ Human connectedness | 0.007 (-0.087 – 0.099) | 0.881 | — | — | 0.007 (-0.087 – 0.099) | 0.881 |
| ⇨ Condition | **0.122 (0.034 – 0.213)** | **0.008** | — | — | **0.122 (0.034 – 0.213)** | **0.008** |
| **Gender ⇨** |  |  |  |  |  |  |
| ⇨ Past-negative | 0.008 (-0.091 – 0.103) | 0.868 | — | — | 0.008 (-0.091 – 0.103) | 0.868 |
| ⇨ Past-positive | -0.003 (-0.092 – 0.090) | 0.952 | — | — | -0.003 (-0.092 – 0.090) | 0.952 |
| ⇨ Present-hedonism | **-0.117 (-0.188 – -0.043)** | **0.002** | — | — | **-0.117 (-0.188 – -0.043)** | **0.002** |
| ⇨ Present-fatalism | -0.008 (-0.105 – 0.092) | 0.877 | — | — | -0.008 (-0.105 – 0.092) | 0.877 |
| ⇨ Future | 0.067 (-0.027 – 0.163) | 0.166 | — | — | 0.067 (-0.027 – 0.163) | 0.166 |
| ⇨ Evaluation | -0.049 (-0.140 – 0.045) | 0.301 | — | — | -0.049 (-0.140 – 0.045) | 0.301 |
| ⇨ Human connectedness | 0.048 (-0.045 – 0.142) | 0.319 | — | — | 0.048 (-0.045 – 0.142) | 0.319 |
| ⇨ Condition | -0.033 (-0.127 – 0.057) | 0.481 | — | — | -0.033 (-0.127 – 0.057) | 0.481 |
| Past-negative WITH past-positive | -0.033 (-0.156 – 0.088) | 0.604 | — | — | — | — |
| Past-negative WITH present-hedonism | **0.194 (0.091 – 0.288)** | **<0.001** | — | — | — | — |
| Past-negative WITH present-fatalism | **0.139 (0.028 – 0.245)** | **0.011** | — | — | — | — |
| Past-negative WITH future | -0.118 (-0.227 – 0.000) | 0.043 | — | — | — | — |
| Past-positive WITH present-hedonism | **0.205 (0.086 – 0.319)** | **0.001** | — | — | — | — |
| Past-positive WITH present-fatalism | **0.121 (0.005 – 0.231)** | **0.036** | — | — | — | — |
| Past-positive WITH future | **0.140 (0.013 – 0.269)** | **0.033** | — | — | — | — |
| Present-hedonism WITH present-fatalism | **0.144 (0.038 – 0.249)** | **0.008** | — | — | — | — |
| Present-hedonism WITH future | -0.069 (-0.195 – 0.068) | 0.293 | — | — | — | — |
| Present-fatalism WITH future | -0.110 (-0.217 – 0.001) | 0.048 | — | — | — | — |
| Evaluation WITH human connectedness | **0.668 (0.616 – 0.713)** | **<0.001** | — | — | — | — |
| Evaluation WITH condition | **0.389 (0.297 – 0.467)** | **<0.001** | — | — | — | — |
| Human connectedness WITH condition | **0.388 (0.291 – 0.474)** | **<0.001** | — | — | — | — |
| Past-negative: **R^2^ = 6.4% (p = 0.011)**  Past-positive: **R^2^ = 7.9% (p = 0.011)**  Present-hedonism: **R^2^ = 39.7% (p < 0.011)**  Present-fatalism: R^2^ = 4.1% (p = 0.053)  Future: **R^2^ = 12.1% (p < 0.001)**  Evaluation: **R^2^ = 23.1% (p < 0.001)**  Human connectedness: **R^2^ = 21.2% (p < 0.001)**  Condition: **R^2^ = 21.2% (p < 0.001)** | | |  |  |  |  |
| *Note:* Bold: significant result; Bold and Italic: significant result according to the 95% bootstrapped confidence interval (despite the p-value); β: standardized coefficient; in brackets: 95% bootstrapped confidence intervals of β; R^2^: explained variance | | | | | | |
